# Supplementary material for: Admission to psychiatric hospital for mental illnesses 2 years prechildbirth and postchildbirth in Scotland: a health informatics approach to assessing mother and child outcomes
Source: BMJ Open. 2017 Sep 1;7(9):e016908. doi: 10.1136/bmjopen-2017-016908 (PMC5588979; doi:10.1136/bmjopen-2017-016908)
Supplement: Author's manuscript [file bmjopen-2017-016908.draft_revisions.pdf]

BMJ Open is committed to open peer review. As part of this commitment we make the peer review history of every article we publish publicly available.

When an article is published we post the peer reviewers' comments and the authors' responses online. We also post the versions of the paper that were used during peer review. These are the versions that the peer review comments apply to.

The versions of the paper that follow are the versions that were submitted during the peer review process. They are not the versions of record or the final published versions. They should not be cited or distributed as the published version of this manuscript.

BMJ Open is an open access journal and the full, final, typeset and author-corrected version of record of the manuscript is available on our site with no access controls, subscription charges or pay-per-view fees (http://bmjopen.bmj.com).

If you have any questions on BMJ Open's open peer review process please email editorial.bmjopen@bmj.com

Admission to Psychiatric Hospital for mental illnesses 2 years pre and post childbirth in Scotland: a health informatics approach to assessing mother and child outcomes.

Journal:	*BMJ Open*
Manuscript ID	bmjopen-2017-016908
Article Type:	Research
Date Submitted by the Author:	21-Mar-2017
Complete List of Authors:	Langan, Julie; University of Glasgow, Institute of Health and Wellbeing<br>McLean, Gary; University of Glasgow, Institute of Health and Wellbeing<br>Martin, Daniel; Glasgow University, Institute of Health and Wellbeing<br>Cantwell, Roch; Leverndale Hospital, Perinatal Mental Health Services<br>Smith, Daniel; University of Glasgow, Institute of Health and Wellbeing
**Primary Subject Heading**:	Mental health
Secondary Subject Heading:	Health informatics, Health services research, Obstetrics and gynaecology, Public health
Keywords:	post partum period, mental illness, child outcomes, Mother and Baby Unit, admission, readmission

For peer review only

SCHOLARONE™
Manuscripts

Admission to Psychiatric Hospital for mental illnesses 2 years pre and post childbirth in Scotland: a health informatics approach to assessing mother and child outcomes.

Julie Langan Martin[1], Gary McLean[1], Daniel Martin[1], Roch Cantwell[2], Daniel J. Smith[1].

[1] Institute of Health and Wellbeing, College of Medical, Veterinary and Life Sciences, University of Glasgow, Mental Health and Wellbeing, Gartnavel Royal Hospital, 1055 Great Western Road, Glasgow G12 0XH

[2] Perinatal Mental Health Service & West of Scotland Mother & Baby Unit, Leverndale Hospital, 510 Crookston Road, Glasgow G53 7TU

Corresponding Author Julie Langan Martin Institute of Health and Wellbeing, College of Medical, Veterinary and Life Sciences, University of Glasgow, Mental Health and Wellbeing, Gartnavel Royal Hospital, 1055 Great Western Road, Glasgow G12 0XH Phone No: +44 (0)141 211 3927 Fax :+44 (0) 141 211 0356 Email: julie.langan@glasgow.ac.uk

KEYWORDS: **post partum period, mental illness, child outcomes, Mother and Baby Unit, admission and readmission**

WORD COUNT: ABSTRACT: 300 MAIN TEXT: 2,519

For peer review only

ABSTRACT

OBJECTIVE: To identify factors associated with: admission to a specialist Mother and Baby Unit (MBU), readmission to psychiatric hospital and the impact of perinatal mental illness on early childhood development using a data linkage approach in the 2 years pre and post childbirth.

METHODS: Scottish maternity records (SMR02) were linked to psychiatric hospital admissions (SMR04). 3,290 pregnancy-related psychiatric admissions for 1,730 women were assessed. To investigate factors associated with MBU admission, the group of mothers admitted to a MBU were compared to those admitted to general psychiatric wards. To evaluate factors associated with future readmission, a group of mothers with a history of psychiatric admission were compared to those without a previous admission. To assess the impact of perinatal mental illness on early child development, a pragmatic indicator for 'high risk of impaired development', defined as a child who was recorded as requiring intensive treatment at any time under the health plan indicators (HPI) and/or who had no record of completing three doses of the 5 in 1 vaccine by 12 months was generated.

RESULTS: Women with a pregnancy-related psychiatric admission, who had a prior psychiatric admission were over two and a half times more likely to have at least one readmission during the two year follow-up period (OR:2.59, 95%CI 2.09-3.20). In total, 190 (11.0%) were admitted to a MBU. Women admitted to a MBU were more likely than those admitted to general psychiatric wards to come from affluent areas and were from older age groups. Almost one third (29%) of children born to mothers with a pregnancy-related psychiatric admission were assessed as high risk of developmental impairments.

CONCLUSIONS: A health informatics approach has potential for improving understanding of social and clinical factors which contribute to the outcomes of perinatal mental illness, as well as adverse developmental outcomes for offspring.

For peer review only - http://bmjopen.bmj.com/site/about/guidelines.xhtml

For peer review only

Strengths and limitations of this study

- Whole of Scotland childbirth and psychiatric admission data used for analyses, rather than local data only.
- Robust measure of socioeconomic status (Scottish Index of Multiple Deprivation) compared with other studies which have used educational status.
- These analyses used only psychiatric admission data, rather than outpatient psychiatric attendances.
- Our definition of 'high risk of developmental impairment' in offspring was a composite and pragmatic measure derived from the limited child health outcome data and there has been some inconsistency in the implementation of this across health boards.

For peer review only

For peer review only - http://bmjopen.bmj.com/site/about/guidelines.xhtml

BACKGROUND:

Good maternal mental health is important for normal childhood development (Stein et al., 2014). Maternal mental illness can disrupt optimal parenting processes, and can adversely affect childhood development, especially emotional development (Leis et al., 2013; Yarrow et al., 1984). In particular maternal mental illness may affect children's executive functioning (Hughes et al., 2013), and may be associated with increased rates of childhood depression (Murray et al., 2011). Maternal depression both during pregnancy and in the post-natal period is common and is estimated to affect between 10 and 15% of women (O'Hara and Swain,1996; Peralstein et al., 2009). While the prevalence of postpartum psychosis is relatively low at 1/1000 births (Terp and Mortensen, 1998), it is known to be associated with severe adverse outcomes, including maternal suicide and infanticide (Wisner et al., 1994).

The joint admission of mentally ill mothers and their infants was pioneered by Thomas Main in 1948 (Brockington, 1996). Since then the UK has acted as a leader in this field (Cazas and Glangeaud-Freudenthal, 2004). Currently in the UK where possible, postpartum women (and those in later pregnancy) with severe mental illnesses such as psychosis or severe depressive disorder are admitted to a specialised Mother and Baby Unit (MBU). The current Scottish Intercollegiate Guidelines Network (SIGN) clinical guidelines for the management of perinatal mood disorder reflect this (SIGN, 2012) . MBUs are highly specialised, expensive and limited resources. Although there are currently 15 in England and 2 in Scotland (Royal College of Psychiatrists Quality Network for Perinatal Mental Health Services, 2016), access to this specialised service is poorer in many other High Income Countries (HICs; such as the US and Canada) and Low And Middle Income Countries (LAMICs). Given the importance of adverse childhood experiences (ACEs) in future health (Bellis et al., 2015, Felitti et al., 1998), perinatal mental illness and the potential impact it has on offspring is a priority area for research and practice.

AIMS OF THE STUDY:

The aim of the study was to use a data linkage approach to investigate factors associated with admission to a specialist Mother and Baby Unit (MBU) and factors associated with risk of future readmission in the 2 years pre and post childbirth. Finally, the impact of perinatal mental illness on the early childhood development of offspring was examined.

For peer review only - http://bmjopen.bmj.com/site/about/guidelines.xhtml

MATERIALS AND METHODS:

We used a dataset from the Information Services Division (ISD) of NHS Scotland which included all perinatal records (SMR02) between 2005-2009, linked to all psychiatric hospital admission records (SMR04) between 2003-2011. This dataset has been described elsewhere (Langan Martin et al., 2014). Early childhood developmental outcomes (assessed by a Health Visitor at both 10-day and 6-8 week child-health checks) were also available. In total, 3,290 pregnancy-related psychiatric admissions for 1,730 women were assessed. For deliveries in 2005, psychiatric admissions between 2003 (two years before) and 2007 (two years after) were captured. Similarly, for deliveries in 2009, psychiatric admissions from 2007 (two years before) until 2011 (two years after) were captured. For each maternity record, any psychiatric admission was reported by week for the 2 year pre-and post-childbirth periods. All statistical analyses were performed in STATA version 13.1 (STATA).

To investigate factors associated with admission to the MBU, we compared the group of mothers admitted to a MBU unit to those admitted to a general psychiatric ward up to 2 years post-partum on a range of sociodemographic characteristics (age, social deprivation and previous pregnancy). We also included the geographical location (health board area) of the mother.

To explore possible factors associated with future readmission, we compared a group of mothers with a history of prior psychiatric admission to those without a history of admission two or more years prior to the index admission. We compared those with and without a previous admission by diagnosis, Scottish Index of Multiple Deprivation (SIMD) quintile, age group, length of stay, whether the mother had at least one readmission, time of admission and whether the mother had a previous pregnancy. We used Cox regression to describe the association between each variable and the risk of admission between those with a history of prior psychiatric admission and those without. Where possible, odds ratios were adjusted for age and deprivation quintile.

Finally to assess the impact of perinatal mental illness on early child development, we generated a pragmatic indicator for ‘high risk of impaired development’, defined as a child who was recorded as requiring intensive treatment at any time under the health plan indicators (HPI) and/or who had no record of completing three doses of the 5 in 1 vaccine by 12 months. The HPI is assessed at first visit, at six to eight weeks. For the HPI three possible outcomes can be recorded by the Health Visitor for the level of care required (‘core’, ‘additional’ and ‘intensive’). It should be noted that there has been inconsistency in the implementation of the HPI across health boards, so results should be interpreted cautiously.

For peer review only - http://bmjopen.bmj.com/site/about/guidelines.xhtml

RESULTS:

In total 190 (11.0%) of this sample were admitted to a specialist MBU in Scotland. Table 1 highlights some important differences between MBU versus non-MBU admissions. Firstly, women admitted to an MBU were significantly more likely to be admitted with non-affective psychosis (OR = 1.97, 95%CI 1.22-3.18), affective psychosis (OR = 2.44, 95%CI 1.37-4.33) and non-psychotic depressive episodes (OR = 1.93, 95%CI 1.42-1.63). It is also notable that women admitted to an MBU were significantly more likely to live within affluent areas (and less likely to come from deprived areas) and in general more likely to be from an older age group (31-35, 36-40 and over 40) (Table 1). Women admitted to an MBU were largely from the NHS Greater Glasgow and Clyde Health Board area (54.2% overall).

Table 1 also shows that MBU admissions were significantly less likely to have had a previous pregnancy and less likely to have had a previous psychiatric admission.

In total 562 (32.5%) of the 1,730 women had a history of prior psychiatric admission. Table 2 shows that this group were more likely to be admitted with a diagnosis of affective psychosis (7.3% vs. 2.6%, adjusted OR = 3.00, 95%CI 1.82-4.94) and non-affective psychosis (10.0% vs. 6.4%, adjusted OR = 1.55, 95%CI 1.07-2.25)but less likely to have an admission for a postpartum or non-psychotic depressive episode diagnosis. Those with a previous history of psychiatric admission were also significantly more likely to be located in the two most deprived quintiles and less likely to come from the two most affluent quintiles. Table 2 also shows they were less likely to be under 25 but significantly more likely to be from the 31-35 and 36-40 age groups. Those with a previous psychiatric admission were significantly more likely to have at least one readmission (54.6% vs. 31.5%, adjusted OR = 2.59, 95%CI 2.09-3.20). No significant differences were identified for length of stay, previous pregnancy or time of admission.

Table 3 highlights important differences in length of stay for MBU versus non-MBU admissions. It shows that women admitted to an MBU were significantly less likely to have brief stays in hospital (of 10 days or less) but significantly more likely to have stays of more than 10 days, and more than twice as likely to have had a stay of 21-40 days or 40 days or more. Table 3 also shows that although those women who attended an MBU and had a brief (less than 5 days) admission were less likely to have been readmitted, the opposite was true for those with longer admissions (21-40 days or 40 days or more). Longer admission appeared therefore to predict readmission for those who attended an MBU.

For peer review only - http://bmjopen.bmj.com/site/about/guidelines.xhtml

Table 4 shows that 518 (29.9%) of offspring were defined as being at 'high risk of developmental impairment' according to our criteria. Those at 'high risk' were more likely (although not statistically significantly) to have had a mother with non-affective psychosis (OR = 1.37, 95%CI 0.94-2.01) and significantly less likely to have a mother with a diagnosis of non-psychotic depression (OR = 0.65, 95%CI 0.51-0.83). It is notable that they were significantly more likely to come from the most deprived quintiles and less likely to be from more affluent quintiles. No differences were found by age of mother. Those children identified as 'high risk' were more likely to have had a mother with a history of a previous psychiatric admission and more likely to be readmitted during the assessment period.

DISCUSSION:

The aim of this study was to assess the extent to which sociodemographic factors and perinatal mental illness affects treatment and outcomes for both mothers and children. We covered three main areas: impact of prior psychiatric admission on future admissions, the role of MBUs and factors associated with adverse child development outcomes.

In this large sample of Scottish women with a childbirth-related psychiatric admission, those with a record of prior psychiatric admission (compared to those with no history of admission) were more than two and a half times more likely to have at least one readmission during the two-year study follow-up period. They also had a different pattern of admission diagnosis: they were more likely to be admitted with a diagnosis of affective psychosis (predominantly bipolar disorder) and non-affective psychosis (predominantly schizophrenia) but less likely to have an admission for a non-psychotic depressive diagnosis. In line with previous work, this confirms that childbirth represents a period of elevated risk for relapse for women known to have serious psychiatric disorders such as bipolar disorder and schizophrenia (Terp and Mortensen, 1998;Kendell et al., 1987).

There was also an important difference between those with and without a previous psychiatric admission in terms of socioeconomic deprivation. Women with a previous history of psychiatric admission were more likely to live in the two most deprived quintiles and less likely to come from the two most affluent quintiles. Studies investigating the link between deprivation and admission rate are limited. Some authors have described a link between psychiatric diagnosis, deprivation and admission rate (Harrison et al., 1995) and others have suggested a link between socio-economic status and admission to forensic psychiatry services (Coid et al., 2001). Further more detailed investigation in this area is required.

For peer review only - http://bmjopen.bmj.com/site/about/guidelines.xhtml

For peer review only

In this study, we compared MBU admissions with admissions to a general psychiatric ward on a range of demographic and clinical variables. An important finding was that women admitted to a MBU were significantly more likely to come from socially affluent areas (and less likely to come from socially deprived areas) and in general were more likely to be from the older age groups. It is possible that this might reflect a health inequality in terms of access to MBU admission but this is a question which requires further research. To date literature exploring accessibility to MBUs is limited. There is only one systematic review investigating outcomes for women admitted to a mother and baby unit (Gillham and Wittkowski, 2015). In this study accessibility to MBU was not reported and no objective marker of deprivations (such as SIMD) was included (Gillham and Wittkowski, 2015).

We found that admissions for those admitted to an MBU tended to be longer. This maybe a consequence of a more severe presentation requiring more intensive support or may be related to the need for a higher level of social functioning to ensure safe care for mothers and their babies on discharge from hospital. Longer length of stay in an MBU compared to a general psychiatric ward was also reported by Cantwell et al., (2002). However, in our study, we also found that readmission rates for those with longer stays were significantly higher for MBU attendees. Interestingly other studies have reported that “average length of stay has been systematically found to be negatively associated with readmission risk/rates” within a general adult psychiatric setting (Kalseth et al., 2016).

Several notable findings arose from our comparison of mothers of children identified as at ‘high risk of developmental impairment’. Firstly, almost one third (29%) of children fell into this category. This rate is similar to a study by Whitmore et al., (2011) who reported that social services were involved in 31.6% of all admissions to their MBU in Birmingham and 10% of admissions resulted in separate discharges (Whitmore et al., 2011). Secondly, our findings indicate that children in the ‘high risk’ group were more likely to come from deprived locations, have mothers with a previous psychiatric admission and have had a mother admitted with a non-affective psychosis (schizophrenia). This finding is again similar to that by Whitmore et al., (2011) who reported that women with a diagnosis of schizophrenia were discharged separately more often than other groups. However Whitmore et al., (2011) did not report deprivation status or previous psychiatric admission. Taken together, our findings suggest that there may be some benefit to identifying mothers at particularly high risk in terms of adverse developmental outcomes for their children, such as mothers living in more

For peer review only - http://bmjopen.bmj.com/site/about/guidelines.xhtml

deprived communities who are known to have a history of previous psychiatric admissions for schizophrenia.

STRENGTHS AND LIMITATIONS:

Strengths of this study include the completeness of the sample, which was obtained from record linkage for the whole of Scotland. However, some limitations in this work are acknowledged. Firstly, only psychiatric admission data were used, with no use of out-patient data. Our findings are therefore focused on the more severe end of the mental illness spectrum. Although we were able to determine if individuals had had a previous psychiatric admission, information about the number of previous admissions, family support, or access to Crisis teams were not available. The comparisons of women admitted to MBUs with women admitted to general psychiatric wards needs to be interpreted cautiously. This is particularly in relation to the timing of admissions in relation to childbirth, as women will only be admitted to MBUs in the very late stages of pregnancy and in the first year post-partum. The general psychiatric admissions included admissions up to 2 years pre partum and 2 years post-partum. Limited information about time and length stay at an MBU prevented any cost analysis being undertaken. Our definition of 'high risk of developmental impairment' in offspring was a composite and pragmatic measure derived from the limited child health outcome data which was available to us from record linkage. There has also been some inconsistency in the implementation of the HPI across health boards. A more detailed and comprehensive assessment of child development in this group of mothers is therefore warranted.

CONCLUSION:

In conclusion, this study found that a health informatics/data linkage approach has considerable potential for improving our understanding of the social and clinical factors which contribute to perinatal mental illness in mothers in Scotland, as well as adverse developmental outcomes for their children. To date there has been no systematic assessment of the benefits (or adverse effects) of specialised MBUs in Scotland. Although this study identified that MBUs appear to be looking after women with more severe psychiatric disorders in the period soon after childbirth, there was also the possibility of a health inequality in terms of access to MBUs (specifically that mothers living in more deprived areas and younger mothers may not be accessing MBU admission as often as might be expected).

For peer review only - http://bmjopen.bmj.com/site/about/guidelines.xhtml

Table 1. Admissions to Mother Baby Unit (MBU) versus general psychiatric ward admissions

	MBU admission	Non-MBU admission	Odds ratio, adjusted by age and deprivation (95%CI)
	Number (%)	Number (%)	
Total	190 (11.0)	1,539 (89)	
**Diagnosis**			
Non affective psychosis	24 (12.6)	107 (7.0)	1.97 (1.22-3.18)
Affective psychosis	18 (9.5)	53 (3.4)	2.44 (1.37-4.33)
Post-partum (0-12 weeks) psychosis	18 (9.5)	87 (5.7)	1.56 (0.91-2.70)
Non psychotic depressive episodes	85 (44.7)	447 (29.0)	1.93 (1.42-1.63)
Any other diagnosis	130 (68.4)	1,292 (84.0)	0.45 (0.32-0.63)
**Deprivation Quintile**			
Most deprived	64 (33.7)	656 (43.4)	0.68 (0.49-0.93)
2	34 (17.9)	336 (22.2)	0.76 (0.51-1.13)
3	28 (14.7)	246 (16.3)	0.88 (0.58-1.31)
4	37 (19.5)	169 (10.2)	2.00 (1.35-2.96)
Least deprived	27 (14.2)	105 (6.9)	2.33 (1.49-3.65)
**Age group**			
Under 20	16 (8.4)	172 (11.2)	0.78 (0.45-1.34)
20-25	40 (21.1)	465 (30.2)	0.65 (0.45-0.95)
26-30	47 (24.7)	407 (26.4)	0.90 (0.63-1.29)
31-35	50 (26.3)	293 (19.0)	1.38 (0.97-1.96)
36-40	31 (16.3)	170 (11.0)	1.49 (1.03-1.58)
Over40	6 (3.2)	32 (2.1)	1.55 (0.63-3.80)
**Geographical location**			
Greater Glasgow&Clyde	103 (54.2)	389 (25.3)	3.50 (2.57-4.76)
Lothian	55 (29.0)	169 (11.0)	3.30 (2.32-4.96)
Lanarkshire	12 (6.3)	169 (11.0)	0.54 (0.29-1.00)
Any other Health Board	20 (10.5)	812 (52.7)	0.02 (0.01 to 0.03)
With at least one readmission	64 (33.7)	610 (39.6)	0.81 (0.56-1.06)
Had previous pregnancy	95 (50.0)	935 (60.8)	0.57 (0.41-0.78)
History of prior psychiatric admission	43 (22.6)	519 (33.7)	0.57 (0.40-0.83)
Time of first admission			
Post-partum (0-12-weeks	102 (53.7)	125 (8.1)	11.96 (8.4-16.8)

Note: women could have more than one diagnosis so numbers are greater than the number of women. In total 27 women had no SIMD quintile assigned due to missing or incorrect postcode

For peer review only - http://bmjopen.bmj.com/site/about/guidelines.xhtml

Table 2. Characteristics of those with history of prior psychiatric admission versus those without

	Previous admission<br>Number (% of total)	No previous admission<br>Number (% of total)	Odds ratio adjusted by age and deprivation (95%CI)
Total	562 (32.5)	1,168 (67.5)	
**Diagnosis**			
Non affective psychosis	56 (10.0)	75 (6.4)	1.55 (1.07-2.25)
Affective psychosis	41 (7.3)	30 (2.6)	3.00 (1.82-4.94)
Post-partum psychosis	57 (10.1)	170 (14.6)	0.36 (0.20-0.64)
Non psychotic depressive episodes	137 (24.4)	395 (33.8)	0.63 (0.50-0.79)
Any other diagnosis	450 (80.1)	973 (83.3)	0.78 (0.60-1.02)
**Deprivation Quintile**			
Most deprived	251 (45.7)	470 (40.7)	1.31 (1.07-1.61)
2	131 (23.9)	239 (20.7)	1.11 (0.87-1.42)
3	85 (15.5)	189 (16.4)	0.90 (0.68-1.20)
4	54 (9.8)	152 (13.2)	0.68 (0.49-0.95)
Least deprived	28 (5.1)	104 (9.0)	0.48 (0.31-0.75)
**Age group**			
Under 20	37 (6.6)	152 (13.0)	0.44 (0.30-0.64)
20-25	146 (26.0)	359 (30.7)	0.78 (0.62-0.98)
26-30	160 (28.5)	294 (25.2)	1.19 (0.95-1.50)
31-35	126 (22.4)	217 (18.6)	1.31 (1.01-1.68)
36-40	80 (14.2)	121 (10.4)	1.45 (1.07-1.97)
Over40	13 (2.3)	25 (2.1)	1.01 (0.50-2.03)
**Length of stay**			
Five days or less	203 (36.1)	472 (40.4)	0.87 (0.70-1.09)
6-10 days	112 (19.9)	234 (20.0)	0.99 (0.76-1.28)
11-20 days	104 (18.5)	212 (18.2)	0.95 (0.73-1.24)
21-40 days	71 (12.6)	150 (12.8)	0.99 (0.73-1.26)
40 plus days	72 (12.8)	100 (8.6)	1.50 (1.07-2.09)
With at least one readmission	307 (54.6)	368 (31.5)	2.59 (2.09-3.20)
Had previous pregnancy	359 (63.9)	671 (57.5)	1.03 (0.82-1.29)
Time of first admission			
Post-partum (0-12-weeks	57 (10.1)	170 (14.6)	0.71 (0.48-1.02)

Note: women could have more than one diagnosis so numbers are greater than the number of women. In total 27 women had no SIMD quintile assigned due to missing or incorrect postcode

For peer review only - http://bmjopen.bmj.com/site/about/guidelines.xhtml

Table 3. Comparison of MBU versus non-MBU admissions

	MBU admission<br>Number (%)	Non-MBU admission<br>Number (%)	Odds ratio adjusted by age and deprivation (95%CI)
**Length of admission**			
Five days or less	35 (18.4)	640 (41.6)	0.34 (0.23-0.50)
6-10 days	25 (13.2)	320 (20.8)	0.59 (0.39-0.92)
11-20 days	42 (22.1)	274 (17.8)	1.27 (0.86-1.85)
21-40 days	49 (25.8)	172 (11.2)	2.51 (1.73-3.63)
40 plus days	39 (20.4)	133 (8.6)	2.48 (1.65-3.71)
**Readmission rates**			
Five days or less	12 (18.8)	218 (35.7)	0.41 (0.21-0.79)
6-10 days	10 (15.6)	133 (21.8)	0.66 (0.32-1.33)
11-20 days	12 (18.8)	121 (19.8)	0.93 (0.48-1.80)
21-40 days	15 (23.4)	77 (12.6)	2.11 (1.13-3.96)
40 plus days	15 (23.4)	61 (10.0)	2.75 (1.45-5.20)

For peer review only

For peer review only - http://bmjopen.bmj.com/site/about/guidelines.xhtml

Table 4. Characteristics of mothers with children defined as being at 'high risk of developmental impairment'

	At risk	Not at risk	Odds ratio adjusted by age and deprivation (95%CI)
	Number (% of total)	Number (% of total)	
Total	518 (29.9)	1,212 (70.1)	
**Diagnosis**			
Non affective psychosis	49 (9.5)	82 (6.7)	1.37 (0.94-2.01)
Affective psychosis	22 (4.3)	49 (4.0)	1.24 (0.73-2.12)
Post-partum psychosis	21 (4.1)	84 (6.9)	0.62 (0.37-1.03)
Non psychotic depressive episodes	127 (24.5)	405 (33.4)	0.65 (0.51-0.83)
Any other diagnosis	426 (82.2)	997 (82.3)	0.93 (0.70-1.22)
**Deprivation Quintile**			
Most deprived	274 (54.0)	448 (37.5)	1.88 (1.52-2.32)
2	112 (22.1)	227 (21.5)	1.03 (0.80-1.33)
3	61 (12.0)	214 (17.9)	0.61 (0.45-0.84)
4	36 (7.1)	169 (14.1)	0.46 (0.32-0.68)
**Least deprived**	24 (7.3)	108 (9.3)	0.51 (0.32-0.81)
Age group			
Under 20	61 (11.8)	128 (10.6)	1.02 (0.73-1.45)
20-25	154 (29.7)	351 (29.0)	0.91 (0.72-1.15)
26-30	154 (29.7)	300 (24.8)	1.32 (0.98-1.67)
31-35	90 (17.4)	253 (20.9)	0.90 (0.69-1.17)
36-40	47 (9.1)	154 (12.7)	0.73 (0.51-1.03)
Over40	12 (2.3)	26 (2.2)	1.15 (0.56-2.33)
**Length of stay**			
Five days or less	213 (41.1)	456 (37.6)	1.16 (0.93-1.44)
6-10 days	103 (19.9)	244 (20.1)	0.91 (0.70-1.19)
11-20 days	93 (18.0)	223 (18.4)	0.98 (0.73-1.29)
21-40 days	48 (11.2)	166 (13.7)	0.84 (0.60-1.17)
40 plus days	51 (9.9)	123 (10.2)	0.98 (0.68-1.40)
With at least one readmission	235 (45.4)	440 (36.3)	1.35 (1.08-1.67)
Had previous pregnancy	319 (61.6)	711 (58.9)	1.11 (0.89-1.40)
History of prior psychiatric admission	215 (41.5)	347 (28.6)	1.76 (1.41-2.21)
Time of first admission			
Post-partum (0-12-weeks	73 (14.1)	154 (12.7)	1.31 (0.96-1.79)

Note: women could have more than one diagnosis so numbers are greater than the number of women. In total 27 women had no SIMD quintile assigned due to missing or incorrect postcode

For peer review only

For peer review only - http://bmjopen.bmj.com/site/about/guidelines.xhtml

Contributor ship statement:
GM undertook the data analyses. JLM and DJM wrote the initial draft of the paper. GM, RC and DJM were involved in the drafting of the paper

Competing interests:
All authors report no competing interests.

Funding:
This project was funded by a CSO Health Informatics Call Grant

Data sharing statement:
Data included in the study are available to the authors only

For peer review only

For peer review only - http://bmjopen.bmj.com/site/about/guidelines.xhtml

REFERENCES:

Bellis MA, Hughes K, Leckenby N Hardcastle KA, Perkins C, Lowey H. Measuring mortality and the burden of adult disease associated with adverse childhood experiences in England: a national survey. J Public Health (Oxf). 2015; 37(3):445-54. doi: 10.1093/pubmed/fdu065. Epub 2014 Aug 30.

Brockington IF. Oxford: Oxford University Press; 1996. Motherhood and Mental Health

Cantwell R, Sisodia N, Oates M. A comparison of mother & baby admissions to a specialist inpatient service with postnatal admissions to generic services. 2002 *Proceedings of the Marce Society Biennial Conference*, Sydney.

Cazas O, Glangeaud-Freudenthal NM-C. The history of Mother-Baby Units (MBUs) in France and Belgium and of the French version of the Marcé checklist. *Archives of Women's Mental Health*. 2004;7(1):53-58. doi:10.1007/s00737-003-0046-0.

Coid J, Kahtan N, Cook A, Gault S, JarmanB. Predicting admission rates to secure forensic psychiatry services. Psychol Med. 2001 Apr;31(3):531-9.

Felitti VJ, Anda RF, Nordenberg D, Williamson DF, Spitz AM, Edwards V, Koss MP, Marks JS. Relationship of childhood abuse and household dysfunction to many of the leading causes of death in adults. The Adverse Childhood Experiences Study. Am J Prev Med. 1998;14:245–258

Gillham R, Wittkowski A. Outcomes for women admitted to a mother and baby unit: a systematic review. *International Journal of Women's Health*. 2015;7:459-476. doi:10.2147/IJWH.S69472.

Harrison J, Barrow S, Creed F.Social deprivation and psychiatric admission rates among different diagnostic groups. Br J Psychiatry. 1995 Oct;167(4):456-62.

Hughes C, Roman G, Hart MJ, Ensor R. Does maternal depression predict young children's executive function? - a 4-year longitudinal study. J Child Psychol Psychiatry. 2013 Feb;54(2):169-77. doi: 10.1111/jcpp.12014. Epub 2012 Nov

Kalseth J, Lassemo E, Wahlbeck K, Haaramo P, Magnussen J. Psychiatric readmissions and their association with environmental and health system characteristics: a systematic review of the literature. *BMC Psychiatry*. 2016;16:376. doi:10.1186/s12888-016-1099-8.

Kendell RE, Chalmers JC, Platz C. Epidemiology of puerperal psychoses. Br J Psychiatry 1987;**150**:662–73. doi:10.1192/bjp.150.5.662

Langan-Martin, J., McLean, G., Cantwell, R., and Smith, D. J. (2016) Admission to psychiatric hospital in the early and late post-partum periods: Scottish national linkage study. *BMJ Open*, 6(1), e008758. (doi:10.1136/bmjopen-2015-008758) (PMID:26733566)

Leis JA, Heron J, Stuart EA, Mendelson T. Associations between maternal mental health and child emotional and behavioral problems: does prenatal mental health matter? J Abnorm Child Psychol 2013; 42: 161–71

Murray L., Halligan S.L., & Cooper P.J. (2010). Effects of postnatal depression on mother-infant interactions, and child development In Wachs B.G., editor. (Ed.), Handbook of infant development (pp. 192–220). Malden: Wiley-Blackwell.

For peer review only - http://bmjopen.bmj.com/site/about/guidelines.xhtml

National Institute for Health and Care Excellence. Antenatal and postnatal mental health: Clinical management and service guidance. NICE guidelines [CG45]. February, 2007. [Last accessed on]. Available from: http://guidance.nice.org.uk/CG45/Guidance .

O'Hara MW, Swain AM Rates and risk of postpartum depression: a meta-analysis Int Rev Psychiatry, 8 (1996), pp. 37–54

Pearlstein T, Howard M, Salisbury A, Zlotnick C. Postpartum depression Am J ObstetGynecol, 200 (2009), pp. 357–364

Royal College of Psychiatrists Quality Network for Perinatal Mental Health Services (2016).http://www.rcpsych.ac.uk/pdf/Annual%20Inpatient%20Report%20Final%20Draft.pdf Accessed 16/03/2017

SIGN 127 Management of perinatal mood disorders Available at: **http://www.sign.ac.uk/pdf/sign127.pdf**Accessed 01/03/2017

Stein A, Pearson RM, Goodman SH, Rapa E, Rahman A, McCallum M, Howard LM, Pariante CMEffects of perinatal mental disorders on the fetus and child. Lancet. 2014 Nov 15;384(9956):1800-19. doi: 10.1016/S0140-6736(14)61277-0. Epub 2014 Nov 14.

STATA: https://www.stata.com/stata-news/news28-4/stata13.1/

Terp IM, Mortensen PB. Post-partum psychoses. Clinical diagnoses and relative risk of admission after parturition. Br J Psychiatry 1998;172:521–6.

Yarrow L.J., MacTurk R.H., Vietze P.M., McCarthy M.E., Klein R.P., McQuinston S. Development course of parental stimulation and its relationship to mastery motivation during infancy. Developmental Psychology. 1984;20:492–503.

Whitmore J, Heron J, Wainscott G.Predictors of parenting concern in a Mother and Baby Unit over a 10-year period. Int J Soc Psychiatry. 2011 Sep;57(5):455-61. doi: 10.1177/0020764010365412. Epub 2010 Apr 13.

Wisner KL, Peindl K, Hanusa BH. Symptomatology of affective and psychotic illnesses related to childbearing. J Affect Disord 1994;30:77–87. doi:10.1016/0165-0327(94)90034-5

For peer review only - http://bmjopen.bmj.com/site/about/guidelines.xhtml

STROBE Statement—checklist of items that should be included in reports of observational studies

	Item No	Page Number discussed
**Title and abstract**	1	(*a*) Page 1
		(*b*) Page 2
**Introduction**		
Background/rationale	2	Page 4
Objectives	3	Page 4
**Methods**		
Study design	4	Page 5
Setting	5	Page 5
Participants	6	(*a*) *Cross-sectional study*—Page 5
		(*b*) *No matching*
Variables	7	Page 5
Data sources/ measurement	8*	Page 5
Bias	9	Page 9
Study size	10	Page 5
Quantitative variables	11	Page 5
Statistical methods	12	(*a*) Page 5
		(*b*) Describe any methods used to examine subgroups and interactions Page 5
		(*c*) Explain how missing data were addressed Page 5
		(*d*) *Cross-sectional study*—If applicable, describe analytical methods taking account of sampling strategy Page 5
		(*e*) Describe any sensitivity analyses: n/a

Continued on next page

For peer review only

**Results**		
Participants	13*	(a) Page 5
		(b) n/a
		(c) N/a
Descriptive data	14*	(a) Page 6
		(b) n/a
		(c) *Cohort study*—Summarise follow-up time (eg, average and total amount) n/a
Outcome data	15*	*Cohort study*—Report numbers of outcome events or summary measures over time
		Case-control study—Report numbers in each exposure category, or summary measures of exposure
		Cross-sectional study—Report numbers of outcome events or summary measures Pages 6 and 7 & Tables 1-4
Main results	16	(*a*) Pages 6 and 7 & Tables 1-4
		(*b*) Report category boundaries when continuous variables were categorized n/a
		(*c*) If relevant, consider translating estimates of relative risk into absolute risk for a meaningful time period – n/a
Other analyses	17	Report other analyses done—eg analyses of subgroups and interactions, and sensitivity analyses Pages 6 and 7 & Tables 1-4
**Discussion**		
Key results	18	Summarise key results with reference to study objectives Page 7
Limitations	19	Discuss limitations of the study, taking into account sources of potential bias or imprecision. Discuss both direction and magnitude of any potential bias Pages 3 and 9
Interpretation	20	Give a cautious overall interpretation of results considering objectives, limitations, multiplicity of analyses, results from similar studies, and other relevant evidence Pages 7-9
Generalisability	21	Discuss the generalisability (external validity) of the study results pages 7-9
**Other information**		
Funding	22	Give the source of funding and the role of the funders for the present study and, if applicable, for the original study on which the present article is based page 14

For peer review only

For peer review only - http://bmjopen.bmj.com/site/about/guidelines.xhtml

BMJ Open

Admission to Psychiatric Hospital for mental illnesses 2 years pre and post childbirth in Scotland: a health informatics approach to assessing mother and child outcomes.

Journal:	*BMJ Open*
Manuscript ID	bmjopen-2017-016908.R1
Article Type:	Research
Date Submitted by the Author:	26-May-2017
Complete List of Authors:	Langan, Julie; University of Glasgow, Institute of Health and Wellbeing<br>McLean, Gary; University of Glasgow, Institute of Health and Wellbeing<br>Martin, Daniel; Glasgow University, Institute of Health and Wellbeing<br>Cantwell, Roch; Leverndale Hospital, Perinatal Mental Health Services<br>Smith, Daniel; University of Glasgow, Institute of Health and Wellbeing
**Primary Subject Heading**:	Mental health
Secondary Subject Heading:	Health informatics, Health services research, Public health
Keywords:	mental illness, child outcomes, Mother and Baby Unit, psychiatric admissions

SCHOLARONE™ Manuscripts

For peer review only

Admission to Psychiatric Hospital for mental illnesses 2 years pre and post childbirth in Scotland: a health informatics approach to assessing mother and child outcomes.

Julie Langan Martin[1], Gary McLean[1], Daniel Martin[1], Roch Cantwell[2], Daniel J. Smith[1].

[1] Institute of Health and Wellbeing, College of Medical, Veterinary and Life Sciences, University of Glasgow, Mental Health and Wellbeing, Gartnavel Royal Hospital, 1055 Great Western Road, Glasgow G12 0XH

[2] Perinatal Mental Health Service & West of Scotland Mother & Baby Unit, Leverndale Hospital, 510 Crookston Road, Glasgow G53 7TU

Corresponding Author Julie Langan Martin Institute of Health and Wellbeing, College of Medical, Veterinary and Life Sciences, University of Glasgow, Mental Health and Wellbeing, Gartnavel Royal Hospital, 1055 Great Western Road, Glasgow G12 0XH Phone No: +44 (0)141 211 3927 Fax :+44 (0) 141 211 0356

Email: Julie.langan@glasgow.ac.uk

KEYWORDS: **mental illness, child outcomes, Mother and Baby Unit and hospital admission**

WORD COUNT: ABSTRACT: 300 MAIN TEXT: 2716

For peer review only

ABSTRACT

OBJECTIVE: To identify factors associated with: admission to a specialist Mother and Baby Unit (MBU), and the impact of perinatal mental illness on early childhood development using a data linkage approach in the 2 years pre and post childbirth.

METHODS: Scottish maternity records (SMR02) were linked to psychiatric hospital admissions (SMR04). 3,290 pregnancy-related psychiatric admissions for 1,730 women were assessed. To investigate factors associated with MBU admission, the group of mothers admitted to a MBU were compared to those admitted to general psychiatric wards. To assess the impact of perinatal mental illness on early child development, a pragmatic indicator for 'at potential risk of adversity', defined as a child who was recorded as requiring intensive treatment at any time under the health plan indicators (HPI) and/or who had no record of completing three doses of the 5 in 1 vaccine by 12 months was generated. Logistic regression models were used to describe the association between each variable and the risk of admission between those with a history of prior psychiatric admission and those without.

RESULTS: Women admitted to an MBU were significantly more likely to be admitted with non-affective psychosis (OR = 1.97, 95%CI 1.22-3.18), affective psychosis (OR = 2.44, 95%CI 1.37-4.33) and non-psychotic depressive episodes (OR = 1.93, 95%CI 1.42-2.63). They were more likely to come from affluent areas (OR: 2.33 95%CI 1.49-3.65). Women with a previous history of psychiatric admission were significantly more likely to be located in the two most deprived quintiles. Almost one third (29%) of children born to mothers with a pregnancy-related psychiatric admission were assessed as "at potential risk of adversity."

CONCLUSIONS: A health informatics approach has potential for improving understanding of social and clinical factors, which contribute to the outcomes of perinatal mental illness, as well as potential adverse developmental outcomes for offspring.

For peer review only - http://bmjopen.bmj.com/site/about/guidelines.xhtml

Strengths and limitations of this study

- Whole of Scotland childbirth and psychiatric admission data used for analyses, rather than local data only.
- Robust measure of socioeconomic status (Scottish Index of Multiple Deprivation) compared with other studies, which have used educational status.
- These analyses used only psychiatric admission data, rather than outpatient psychiatric attendances.
- Our definition of 'at potential risk of adversity' in offspring was a composite and pragmatic measure derived from the limited child health outcome data and there has been some inconsistency in the implementation of this across health boards.

For peer review only

For peer review only - http://bmjopen.bmj.com/site/about/guidelines.xhtml

BACKGROUND:

Good maternal mental health is important for normal childhood development[1]. Maternal mental illness can disrupt optimal parenting processes, and can adversely affect childhood development, especially emotional development[2,3,4]. In particular maternal mental illness may be associated with increased rates of childhood depression[5]and may also affect children's executive[6] and cognitive functioning[7]. Maternal depression both during pregnancy and in the post-natal period is common and is estimated to affect between 10 and 15% of women[8,9]. While the prevalence of postpartum psychosis is relatively low at 1/1000 births[10], it is known to be associated with severe adverse outcomes, including maternal suicide and infanticide[11].

The joint admission of mentally ill mothers and their infants was pioneered by Thomas Main in 1948[12]. Since then the UK, Australia and France have acted as leaders in this field[13,14]. Currently in the UK where possible, postpartum women (and those in later pregnancy) with severe mental illnesses such as psychosis or severe depressive disorder are admitted to a specialised Mother and Baby Unit (MBU). In the West of Scotland, women in the post-natal period who are the primary carer of their baby and thought to require psychiatric hospital admission are discussed with (and where possible assessed by) the local Consultant Perinatal Psychiatrist. Hospital admission is arranged if required. While most admissions are voluntary, MBUs can also accommodate women who require compulsory treatment under the Mental Health Act. The current Scottish Intercollegiate Guidelines Network (SIGN) clinical guidelines for the management of perinatal mood disorder reflect this[15]. It is recognised that women in the early postpartum period (0 to 6 weeks after childbirth), are at particularly elevated risk of requiring psychiatric admission[16,17]. In our previous study, we found that this risk remains elevated for up to 2 years post childbirth[16].

MBUs are highly specialised, expensive and limited resources, where expertise in both treatment of psychiatric disorders and childcare are required[18]. Although there are currently 15 in England and 2 in Scotland[19],access to this specialised service is poorer in many other High Income Countries (HICs; such as the US and Canada) and Low And Middle Income Countries (LAMICs) despite women appearing to be satisfied with this type of care[20]. Given the importance of adverse childhood experiences (ACEs) in future health[21,22], perinatal mental illness and the potential impact it has on offspring is a priority area for research and practice.

For peer review only - http://bmjopen.bmj.com/site/about/guidelines.xhtml

AIMS OF THE STUDY:

The aim of the study was to use a data linkage approach to investigate factors associated with admission to a specialist Mother and Baby Unit (MBU) and the impact of perinatal mental illness on early childhood development of offspring.

MATERIALS AND METHODS:

We used a dataset from the Information Services Division (ISD) of NHS Scotland, which included all maternity records (SMR04) between 2005-2009, linked to all psychiatric hospital admission records (SMR02) between 2003-2011. This dataset has been described elsewhere[16]. However in brief, for each maternity record, any psychiatric admission was reported by week for the 104 weeks pre-childbirth and post-childbirth. Admission types were defined by ICD-10 codes: psychosis-only admissions included 'non-affective psychosis' (F20, F20.3, F20.5, F20.6, F20.8, F20.9, F21X, F22.0, F22.8, F22.9, F23.0, F23.1, F23.2, F23.3, F23.8, F23.9, F24X, F28X, F29X), 'affective psychosis' (F25.0, F25.1, F25.2, F25.9, F30.2, F31.2, F31.5, F32.3, F33.3) and 'postpartum psychosis' (F53.0, F53.1, F53.9); admissions due to a non-psychotic depressive episode included F32.0, F32.00, F32.01, F32.1, F32.10, F32.11, F32.2, F32.8, F32.9, F33.0, F33.00, F33.1, F33.10, F33.11, F33.2, F33.4, F33.8, F33.9. For the category of 'other admissions', we included all other ICD-10 codes recorded.

In total, 3,290 pregnancy-related psychiatric admissions for 1,730 women were assessed. For deliveries in 2005, psychiatric admissions between 2003 (two years before) and 2007 (two years after) were captured. Similarly, for deliveries in 2009, psychiatric admissions from 2007 (two years before) until 2011 (two years after) were captured. For each maternity record, any psychiatric admission was reported by week for the 2-year pre-and post-childbirth periods. Early childhood developmental outcomes (assessed by a Health Visitor at both 10-day and 6-8 week child-health checks) were also available. All statistical analyses were performed in STATA version 13.1[23].

To investigate factors associated with admission to the MBU, we compared the group of mothers admitted to a MBU unit to those admitted to a general psychiatric ward up to 2 years post-childbirth on a range of sociodemographic characteristics (age, social deprivation and previous pregnancy). We included the geographical location (health board area) of the mother. We also compared differences in average length of stay.

To explore possible risk factors associated with previous psychiatric admission in the time period covered, we compared a group of mothers with a history of prior psychiatric admission to those

For peer review only - http://bmjopen.bmj.com/site/about/guidelines.xhtml

without a history of admission two or more years prior to the index admission. We compared those with and without a previous admission by diagnosis, Scottish Index of Multiple Deprivation (SIMD) quintile, age group, length of stay, time of admission and whether the mother had a previous pregnancy. SIMD score was used as a measure of social deprivation. The SIMD identifies small areas of multiple deprivation (datazones) across Scotland by combining 38 indicators across 7 domains which are weighted. The domains include: current income (28%), employment (28%), health (14%), education (14%), geographic access to services (9%), crime (5%), and housing (2%) and are weighted based on evidence from Oxford University's Social Disadvantage Research Centre [24]. Individuals were divided into deprivation quintiles, depending on their deprivation score based on national averages.

We used logistic regression to describe the association between each variable and the risk of admission between those with a history of prior psychiatric admission and those without. Odds ratios were adjusted for age and deprivation quintile or for age only (deprivation quintile) and deprivation quintile (age only).

Finally, to assess the impact of perinatal mental illness on early child development, we generated a pragmatic indicator for ''at potential risk of adversity'', defined as a child who was recorded as requiring intensive treatment at any time under the health plan indicators (HPI) and/or who had no record of completing three doses of the 5 in 1 vaccine by 12 months. The HPI is assessed at first visit, at six to eight weeks. For the HPI three possible outcomes can be recorded by the Health Visitor for the level of care required ('core', 'additional' and 'intensive'). It should be noted that results should be interpreted cautiously as there has been inconsistency in the implementation of the HPI across health boards and others have not validated this measure.

RESULTS:

In total 190 (11.0%) of this sample were admitted to a specialist MBU in Scotland. Table 1 highlights some important differences between MBU versus non-MBU admissions. Firstly, women admitted to an MBU were significantly more likely to be admitted with non-affective psychosis (OR = 1.97, 95%CI 1.22-3.18), affective psychosis (OR = 2.44, 95%CI 1.37-4.33) and non-psychotic depressive episodes (OR = 1.93, 95%CI 1.42-2.63). It is also notable that women admitted to an MBU were significantly more likely to live within affluent areas (and less likely to come from deprived areas) and in general more likely to be from an older age group (31-35, 36-40 and over 40) (Table 1). Women admitted to an MBU were largely from the NHS Greater Glasgow and Clyde Health Board area (54.2% overall).

For peer review only - http://bmjopen.bmj.com/site/about/guidelines.xhtml

For peer review only

Table 1 also shows that MBU admissions were significantly less likely to have had a previous pregnancy and less likely to have had a previous psychiatric admission. It also highlights important differences in length of stay for MBU versus non-MBU admissions. It shows that women admitted to an MBU were significantly less likely to have brief stays in hospital (of 10 days or less) but significantly more likely to have stays of more than 10 days, and more than twice as likely to have had a stay of 21-40 days or 40 days or more.

In total 562 (32.5%) of the 1,730 women had a history of prior psychiatric admission. Table 2 shows that this group were more likely to be admitted with a diagnosis of affective psychosis (7.3% vs. 2.6%, adjusted OR = 3.00, 95%CI 1.82-4.94) and non-affective psychosis (10.0% vs. 6.4%, adjusted OR = 1.55, 95%CI 1.07-2.25) but less likely to have an admission for a postpartum or non-psychotic depressive episode diagnosis. Those with a previous history of psychiatric admission were also significantly more likely to be located in the two most deprived quintiles and less likely to come from the two most affluent quintiles. Table 2 also shows they were less likely to be under 25 but significantly more likely to be from the 31-35 and 36-40 age groups. No significant differences were identified for length of stay, previous pregnancy or time of admission.

Table 3 shows that 518 (29.9%) of offspring were defined as being "at potential risk of adversity" according to our criteria. Those at "potential risk" were more likely (although not statistically significantly) to have had a mother with non-affective psychosis (OR = 1.37, 95%CI 0.94-2.01) and significantly less likely to have a mother with a diagnosis of non-psychotic depression (OR = 0.65, 95%CI 0.51-0.83). It is notable that they were significantly more likely to come from the most deprived quintiles and less likely to be from more affluent quintiles. No differences were found by age of mother. Those children identified as "at potential risk of adversity" were more likely to have had a mother with a history of a previous psychiatric admission.

DISCUSSION:

The aim of this study was to assess the extent to which sociodemographic factors and perinatal mental illness affects treatment and outcomes for both mothers and children. We covered two main areas: admissions to an MBU and factors associated with children identified as at "potential risk of childhood adversity".

For peer review only

In this large Scottish sample, women admitted to an MBU (compared to women admitted to general psychiatry wards) were significantly more likely be diagnosed with a psychotic illness (non-affective psychotic illness OR: 1397 95% CI 1.22-3.18 or affective psychotic illness OR: 2.44 95% CI 1.37-4.33). This is in keeping with the notion that MBU admission is reserved for women suffering from the most serious mental disorders[18].

Women admitted to an MBU were also more likely to live within affluent areas (and less likely to come from deprived areas) and in general more likely to be from an older age group (31-35, 36-40 and over 40). It is possible that this might reflect a health inequality in terms of access to MBU admission but this is a question, which requires further research. To date literature exploring accessibility to MBUs is limited. There is one systematic review investigating outcomes for women admitted to a mother and baby unit[25]. Accessibility to an MBU was not reported and no objective marker of deprivation (such as SIMD) was included[25]. Studies investigating the link between deprivation and admission rate are limited. Some authors have described a link between psychiatric diagnoses, deprivation and admission rate[26] while others have suggested a link between socio-economic status and admission to forensic psychiatry services[27].However further detailed investigation in this area is required.

In our study we found that women admitted to an MBU were more likely to have a longer average length of stay (40 plus days OR: 2.48 95% CI 1.65-3.71).This maybe a consequence of a more severe presentation requiring more intensive support or may be related to the need for a higher level of social functioning to ensure safe care for mothers and their babies on discharge from hospital. Further investigation comparing average length of stay in an MBU with particular focus on the cost effectiveness of MBUs may be warranted.

Several notable findings arose from our comparison of mothers of children identified as “at potential risk of adversity”. Firstly, almost one-third (29%) of children fell into this category. This rate is similar to that reported by others. For example, Whitmore and colleagues[28] reported that social services were involved in 31.6% of all admissions to their MBU in Birmingham and 10% of admissions resulted in separate discharges[28]. Howard and colleagues[29] reported that 23% of women were discharged with their babies under some form of social services supervision[29].

Secondly, our findings indicate that children in the “at potential risk of adversity” group were more likely to come from deprived locations, have mothers with a previous psychiatric admission and have

For peer review only - http://bmjopen.bmj.com/site/about/guidelines.xhtml

had a mother admitted with a non-affective psychosis (schizophrenia). This finding is also similar to that by others [18,28,29])who reported that women with a diagnosis of schizophrenia were discharged separately more often than other groups. Although Whitmore and colleague[28] did not report deprivation status, Glangeaud-Freudenthal and colleague [18) and Howard and colleagues (29] have reported that "low social class" was associated with risk of separation [18,29]. Successful parenting is complex, but may be partly associated with family stability and access to financial and social resources[30]. While it is recognised that mothers with severe mental illness, have specific needs relating to their children, their health and social care needs may not always be readily identified by healthcare professionals[31]. In cases of separate discharge, additional support should be made available to minimise distress. Therefore taken together, our findings suggest that there may be some benefit to identifying mothers at particularly high risk in terms of adverse developmental outcomes for their children, such as mothers living in more deprived communities who have a history of previous psychiatric admissions for schizophrenia.

STRENGTHS AND LIMITATIONS:

Strengths of this study include the completeness of the sample, which was obtained from record linkage for the whole of Scotland. However, some limitations in this work are acknowledged. Firstly, only psychiatric admission data were used, with no use of out-patient data. Our findings are therefore focused on the more severe end of the mental illness spectrum. Although we were able to determine if individuals had had a previous psychiatric admission, information about the number of previous admissions, family support, or access to Crisis teams were not available. The comparisons of women admitted to MBUs with women admitted to general psychiatric wards needs to be interpreted cautiously. This is particularly in relation to the timing of admissions in relation to childbirth, as women will only be admitted to MBUs in the very late stages of pregnancy and in the first year post-delivery. The general psychiatric admissions included admissions up to 2 years pre delivery and 2 years post-delivery and may therefore include women with long-term illnesses.

Furthermore, limited information about time and length stay at an MBU prevented any cost analysis being undertaken. Our definition of "at potential risk of adversity" in offspring was a composite and pragmatic measure derived from the limited child health outcome data which was available to us from record linkage. There has also been some inconsistency in the implementation of the HPI across health boards. A more detailed and comprehensive assessment of child development in this group of mothers is therefore warranted.

For peer review only - http://bmjopen.bmj.com/site/about/guidelines.xhtml

CONCLUSION:

In conclusion, this study found that a health informatics/data linkage approach has considerable potential for improving our understanding of the social and clinical factors, which contribute to perinatal mental illness in mothers in Scotland, as well as potential adverse developmental outcomes for their children. To date there has been no systematic assessment of the benefits (or adverse effects) of specialised MBUs in Scotland. Given the current political-economic climate and the importance of early intervention, further research in this area would be of benefit.

For peer review only

For peer review only - http://bmjopen.bmj.com/site/about/guidelines.xhtml

Table 1. Comparison of characteristics of women admitted either with their infant to a Mother-Baby Unit (MBU) or without their infant to a psychiatric ward (Non-MBU) (N=1729)

	MBU admission	Non-MBU admission	Odds ratio, adjusted by age and deprivation (95%CI)
	Number (%)	Number (%)	
Total	190 (11.0)	1,539 (89)	
**Diagnosis**			
Non affective psychosis	24 (12.6)	107 (7.0)	1.97 (1.22-3.18)
Affective psychosis	18 (9.5)	53 (3.4)	2.44 (1.37-4.33)
Post-partum (0-12 weeks) psychosis	18 (9.5)	87 (5.7)	1.56 (0.91-2.70)
Non psychotic depressive episodes	85 (44.7)	447 (29.0)	1.93 (1.42-2.63)
Any other diagnosis	130 (68.4)	1,292 (84.0)	0.45 (0.32-0.63)
**Deprivation Quintile**			
Most deprived (1)	64 (33.7)	656 (43.4)	0.68 (0.49-0.93)
2	34 (17.9)	336 (22.2)	0.76 (0.51-1.13)
3	28 (14.7)	246 (16.3)	0.88 (0.58-1.31)
4	37 (19.5)	169 (10.2)	2.00 (1.35-2.96)
Least deprived (5)	27 (14.2)	105 (6.9)	2.33 (1.49-3.65)
**Age group**			
Under 20	16 (8.4)	172 (11.2)	0.78 (0.45-1.34)
20-25	40 (21.1)	465 (30.2)	0.65 (0.45-0.95)
26-30	47 (24.7)	407 (26.4)	0.90 (0.63-1.29)
31-35	50 (26.3)	293 (19.0)	1.38 (0.97-1.96)
36-40	31 (16.3)	170 (11.0)	1.49 (1.03-1.58)
Over40	6 (3.2)	32 (2.1)	1.55 (0.63-3.80)
**Length of admission**			
Five days or less	35 (18.4)	640 (41.6)	0.34 (0.23-0.50)
6-10 days	25 (13.2)	320 (20.8)	0.59 (0.39-0.92)
11-20 days	42 (22.1)	274 (17.8)	1.27 (0.86-1.85)
21-40 days	49 (25.8)	172 (11.2)	2.51 (1.73-3.63)
40 plus days	39 (20.4)	133 (8.6)	2.48 (1.65-3.71)
**Geographical Location**			
Greater Glasgow and Clyde	103 (54.2)	389 (25.3)	3.50 (2.57-4.76)
Lothian	55 (29.0)	169 (11.0)	3.30 (2.32-4.96)
Lanarkshire	12 (6.3)	169 (11.0)	0.54 (0.29-1.00)
Any other Health Board	20 (10.5)	812 (52.7)	0.02 (0.01 to 0.03)
Had previous pregnancy	95 (50.0)	935 (60.8)	0.57 (0.41-0.78)
History of prior psychiatric admission	43 (22.6)	519 (33.7)	0.57 (0.40-0.83)
Time of first admission			
Post-partum (0-12-weeks)	102 (53.7)	125 (8.1)	11.96 (8.4-16.8)

Note: women could have more than one diagnosis so numbers are greater than the number of women. In total 27 women had no Scottish Index of Multiple Deprivation (SIMD) quintile assigned due to missing or incorrect postcode and were excluded from the regression analysis for all variables

For peer review only - http://bmjopen.bmj.com/site/about/guidelines.xhtml

Table 2. Comparison of characteristics of women with and without a previous psychiatric admission prior to the index admission (N=1720).

	**Previous admission**<br>Number (% of total)	**No previous admission**<br>Number (% of total)	**Odds ratio adjusted by age and deprivation (95%CI)**
Total	562 (32.5)	1,168 (67.5)	
**Diagnosis**			
Non affective psychosis	56 (10.0)	75 (6.4)	1.55 (1.07-2.25)
Affective psychosis	41 (7.3)	30 (2.6)	3.00 (1.82-4.94)
Post-partum psychosis	57 (10.1)	170 (14.6)	0.36 (0.20-0.64)
Non psychotic depressive episodes	137 (24.4)	395 (33.8)	0.63 (0.50-0.79)
Any other diagnosis	450 (80.1)	973 (83.3)	0.78 (0.60-1.02)
**Deprivation Quintile**			
Most deprived	251 (45.7)	470 (40.7)	1.31 (1.07-1.61)
2	131 (23.9)	239 (20.7)	1.11 (0.87-1.42)
3	85 (15.5)	189 (16.4)	0.90 (0.68-1.20)
4	54 (9.8)	152 (13.2)	0.68 (0.49-0.95)
Least deprived	28 (5.1)	104 (9.0)	0.48 (0.31-0.75)
**Age group**			
Under 20	37 (6.6)	152 (13.0)	0.44 (0.30-0.64)
20-25	146 (26.0)	359 (30.7)	0.78 (0.62-0.98)
26-30	160 (28.5)	294 (25.2)	1.19 (0.95-1.50)
31-35	126 (22.4)	217 (18.6)	1.31 (1.01-1.68)
36-40	80 (14.2)	121 (10.4)	1.45 (1.07-1.97)
Over40	13 (2.3)	25 (2.1)	1.01 (0.50-2.03)
**Length of stay**			
Five days or less	203 (36.1)	472 (40.4)	0.87 (0.70-1.09)
6-10 days	112 (19.9)	234 (20.0)	0.99 (0.76-1.28)
11-20 days	104 (18.5)	212 (18.2)	0.95 (0.73-1.24)
21-40 days	71 (12.6)	150 (12.8)	0.99 (0.73-1.26)
40 plus days	72 (12.8)	100 (8.6)	1.50 (1.07-2.09)
Had previous pregnancy	359 (63.9)	671 (57.5)	1.03 (0.82-1.29)
Time of first admission			
Post-partum (0-12-weeks	57 (10.1)	170 (14.6)	0.71 (0.48-1.02)

Note: women could have more than one diagnosis so numbers are greater than the number of women. In total 27 women had no Scottish Index of Multiple Deprivation (SIMD) quintile assigned due to missing or incorrect postcode and were excluded from the regression analysis for all variables

For peer review only

Table 3. Characteristics of mothers with children defined as being at 'potential risk of adversity' (N=1720)

	At potential risk of adversity	Not at potential risk of adversity	Odds ratio adjusted by age and deprivation (95%CI)
	Number (% of total)	Number (% of total)	
**Total**	518 (29.9)	1,212 (70.1)	
MBU Stay	58 (30.4)	133 (69.6)	1.18 (0.84-1.65)
General Ward	459 (29.8)	1,079 (70.2)	1
**Diagnosis**			
Non affective psychosis	49 (9.5)	82 (6.7)	1.37 (0.94-2.01)
Affective psychosis	22 (4.3)	49 (4.0)	1.24 (0.73-2.12)
Post-partum psychosis	21 (4.1)	84 (6.9)	0.62 (0.37-1.03)
Non psychotic depressive episodes	127 (24.5)	405 (33.4)	0.65 (0.51-0.83)
Any other diagnosis	426 (82.2)	997 (82.3)	0.93 (0.70-1.22)
**Deprivation Quintile**			
Most deprived	274 (54.0)	448 (37.5)	1.88 (1.52-2.32)
2	112 (22.1)	227 (21.5)	1.03 (0.80-1.33)
3	61 (12.0)	214 (17.9)	0.61 (0.45-0.84)
4	36 (7.1)	169 (14.1)	0.46 (0.32-0.68)
**Least deprived**	24 (7.3)	108 (9.3)	0.51 (0.32-0.81)
Age group			
Under 20	61 (11.8)	128 (10.6)	1.02 (0.73-1.45)
20-25	154 (29.7)	351 (29.0)	0.91 (0.72-1.15)
26-30	154 (29.7)	300 (24.8)	1.32 (0.98-1.67)
31-35	90 (17.4)	253 (20.9)	0.90 (0.69-1.17)
36-40	47 (9.1)	154 (12.7)	0.73 (0.51-1.03)
Over40	12 (2.3)	26 (2.2)	1.15 (0.56-2.33)
**Length of stay**			
Five days or less	213 (41.1)	456 (37.6)	1.16 (0.93-1.44)
6-10 days	103 (19.9)	244 (20.1)	0.91 (0.70-1.19)
11-20 days	93 (18.0)	223 (18.4)	0.98 (0.73-1.29)
21-40 days	48 (11.2)	166 (13.7)	0.84 (0.60-1.17)
40 plus days	51 (9.9)	123 (10.2)	0.98 (0.68-1.40)
Had previous pregnancy	319 (61.6)	711 (58.9)	1.11 (0.89-1.40)
History of prior psychiatric admission	215 (41.5)	347 (28.6)	1.76 (1.41-2.21)
Time of first admission			
Post-partum (0-12-weeks	73 (14.1)	154 (12.7)	1.31 (0.96-1.79)

Note: women could have more than one diagnosis so numbers are greater than the number of women. In total 27 women had no Scottish Index of Multiple Deprivation (SIMD) quintile assigned due to missing or incorrect postcode and were excluded from the regression analysis for all variables
Note: ''at potential risk of adversity'', is defined as a child who was recorded as requiring intensive treatment at any time under the health plan indicators (HPI) and/or who had no record of completing three doses of the 5 in 1 vaccine by 12 months. HPI is assessed at first visit and at six to eight weeks.

For peer review only

For peer review only - http://bmjopen.bmj.com/site/about/guidelines.xhtml

Contributor ship statement:
GM undertook the data analyses. JLM and DJM wrote the initial draft of the paper. GM, RC and DJM were involved in the drafting of the paper

Competing interests:
All authors report no competing interests.

Funding:
This project was funded by a CSO Health Informatics Call Grant

Data sharing statement:
Data included in the study are available to the authors only

For peer review only

For peer review only - http://bmjopen.bmj.com/site/about/guidelines.xhtml

REFERENCES:

1 Stein A, Pearson RM, Goodman SH, Rapa E, Rahman A, McCallum M, Howard LM, ParianteCMEffects of perinatal mental disorders on the fetus and child.Lancet. 2014 Nov 15;384(9956):1800-19. doi: 10.1016/S0140-6736(14)61277-0. Epub 2014 Nov 14.

2 Milgrom J, Ericksen J, Sved-Williams A. Chapter Impact of Parental Psychiatric Illness on Infant Development in Sutter-Dallay A-L, Glangeaud-Freudenthal NM-C, Guedeney A, Riecher-Rössler A (eds) Joint care of parents and infants in perinatal psychiatry. 2016, Springer,47-78.

3 Leis JA, Heron J, Stuart EA, Mendelson T. Associations between maternal mental health and child emotional and behavioral problems: does prenatal mental health matter? J Abnorm Child Psychol 2013; 42: 161–71

4 Yarrow L.J., MacTurk R.H., Vietze P.M., McCarthy M.E., Klein R.P., McQuinston S. Development course of parental stimulation and its relationship to mastery motivation during infancy. Developmental Psychology. 1984;20:492–503.

5 Murray L., Halligan S.L., & Cooper P.J. Effects of postnatal depression on mother-infant interactions, and child development In Wachs B.G., editor. (Ed.), 2010 Handbook of infant development (pp. 192–220). Malden: Wiley-Blackwell.

6 Hughes C, Roman G, Hart MJ, Ensor R. Does maternal depression predict young children's executive function? - a 4-year longitudinal study. J Child Psychol Psychiatry. 2013 Feb;54(2):169-77. doi: 10.1111/jcpp.12014. Epub 2012 Nov

7 Sutter-Dallay AL, Murray L, Dequae-Merchadou L, Glatigny-Dallay E, Bourgeois ML, Verdoux H.A prospective longitudinal study of the impact of earlypostnatalvs. chronicmaternaldepressive symptoms on child development. Eur Psychiatry.2011 Nov;26(8):484-9. doi: 10.1016/j.eurpsy.2010.05.004. Epub 2010 Jul 10

8 O'Hara MW, Swain AM Rates and risk of postpartum depression: a meta-analysis Int Rev Psychiatry, 1996, 8 :37–54

9 Pearlstein T, Howard M, Salisbury A, Zlotnick C. Postpartum depression Am J ObstetGynecol, 2009. 200: 357–364

10 Terp IM, Mortensen PB. Post-partum psychoses. Clinical diagnoses and relative risk of admission after parturition. Br J Psychiatry 1998;172:521–6.

11 Wisner KL, Peindl K, Hanusa BH. Symptomatology of affective and psychotic illnesses related to childbearing. J Affect Disord 1994;30:77–87. doi:10.1016/0165-0327(94)90034-5

12 Brockington IF. Oxford: Oxford University Press; 1996. Motherhood and Mental Health

13 Brockington I, Butterworth R, Glangeaud-Freudenthal N.An international position paper on mother-infant (perinatal) mental health, with guidelines for clinical practice.Archives of Women's Mental Health. 2017;20(1):113-120. doi:10.1007/s00737-016-0684-7.

For peer review only - http://bmjopen.bmj.com/site/about/guidelines.xhtml

14 Cazas O, Glangeaud-Freudenthal NM-C. The history of Mother-Baby Units (MBUs) in France and Belgium and of the French version of the Marcé checklist. *Archives of Women's Mental Health*. 2004;7(1):53-58. doi:10.1007/s00737-003-0046-0.

15 SIGN 127 Management of perinatal mood disorders Available at: **http://www.sign.ac.uk/pdf/sign127.pdf**Accessed 01/03/2017

16 Langan-Martin, J., McLean, G., Cantwell, R., and Smith, D. J.Admission to psychiatric hospital in the early and late post-partum periods: Scottish national linkage study.*BMJ Open*, 2016 6(1), e008758. (doi:10.1136/bmjopen-2015-008758)(MID:26733566)

17 Munk-Olsen T, Laursen TM, Pedersen CB et al New parents and mental disorders: a population-based registered study. JAMA 2006 296:2582–2589

18 Glangeaud-Freudenthal NM, Howard LM, Sutter-Dallay AL.Treatment - mother-infant inpatient units.Best Pract Res ClinObstetGynaecol. 2014 Jan;28(1):147-57. doi: 10.1016/j.bpobgyn.2013.08.015. Epub 2013 Sep 3.

19 Royal College of Psychiatrists Quality Network for Perinatal Mental Health Services (2016).http://www.rcpsych.ac.uk/pdf/Annual%20Inpatient%20Report%20Final%20Draft.pdf Accessed 16/03/2017

20 Neil S, Sanderson H, Wieck A (2006) A satisfaction survey of women admitted to a psychiatric mother and baby unit in the northwest of England. Arch WomensMent Health 2006: 9:109–112.

21 Bellis MA, Hughes K, Leckenby N Hardcastle KA, Perkins C, Lowey H. Measuring mortality and the burden of adult disease associated with adverse childhood experiences in England: a national survey. J Public Health (Oxf). 2015; 37(3):445-54. doi: 10.1093/pubmed/fdu065. Epub 2014 Aug 30.

22 Felitti VJ, Anda RF, Nordenberg D, Williamson DF, Spitz AM, Edwards V, Koss MP, Marks JS. Relationship of childhood abuse and household dysfunction to many of the leading causes of death in adults. The Adverse Childhood Experiences Study. Am J Prev Med. 1998;14:245–258

23 STATA: https://www.stata.com/stata-news/news28-4/stata13.1/

24 SIMD Scotland http://simd.scotland.gov.uk/publication-2012/introduction-to-simd-2012/overview-of-the-simd/what-is-the-simd/ Accessed 09/05/2017

25 Gillham R, Wittkowski A. Outcomes for women admitted to a mother and baby unit: a systematic review. *International Journal of Women's Health*. 2015;7:459-476. doi:10.2147/IJWH.S69472.

26 Harrison J, Barrow S, Creed F.Social deprivation and psychiatric admission rates among different diagnostic groups. Br J Psychiatry. 1995 Oct;167(4):456-62.

27 Coid J, Kahtan N, Cook A, Gault S, JarmanB.Predicting admission rates to secure forensic psychiatry services. Psychol Med. 2001 Apr;31(3):531-9.

28 Whitmore J, Heron J, WainscottG.Predictors of parenting concern in a Mother and Baby Unit over a 10-year period. Int J Soc Psychiatry. 2011 Sep;57(5):455-61. doi: 10.1177/0020764010365412. Epub 2010 Apr 13.

For peer review only - http://bmjopen.bmj.com/site/about/guidelines.xhtml

29 Howard LM, Thornicroft G, Salmon M, Appleby LPredictors of parenting outcome in women with psychotic disorders discharged from mother and baby units. Acta Psychiatr Scand. 2004 Nov;110(5):347-55.

30 Abel KM, Webb RT, Salmon MP, Wan MW, Appleby L. Prevalence and predictors of parenting outcomes in a cohort of mothers with schizophrenia admitted for joint mother and baby psychiatric care in England. J Clin Psychiatry 2005: 66:781–789.

31 Howard LM, Hunt K The needs of mothers with severe mental illness: a comparison of assessments of needs by staff and patients. Arch Womens Ment Health 2008: 11:131–136.

For peer review only

For peer review only - http://bmjopen.bmj.com/site/about/guidelines.xhtml

STROBE Statement—checklist of items that should be included in reports of observational studies

	Item No	Page Number discussed
**Title and abstract**	1	(*a*) Page 1
		(*b*) Page 2
**Introduction**		
Background/rationale	2	Page 4
Objectives	3	Page 4
**Methods**		
Study design	4	Page 5
Setting	5	Page 5
Participants	6	(*a*) *Cross-sectional study*—Page 5
		(*b*) *No matching*
Variables	7	Page 5
Data sources/ measurement	8*	Page 5
Bias	9	Page 9
Study size	10	Page 5
Quantitative variables	11	Page 5
Statistical methods	12	(*a*) Page 5
		(*b*) Describe any methods used to examine subgroups and interactions Page 5
		(*c*) Explain how missing data were addressed Page 5
		(*d*) *Cross-sectional study*—If applicable, describe analytical methods taking account of sampling strategy Page 5
		(*e*) Describe any sensitivity analyses: n/a

Continued on next page

For peer review only

**Results**		
Participants	13*	(a) Page 5
		(b) n/a
		(c) N/a
Descriptive data	14*	(a) Page 6
		(b) n/a
		(c) *Cohort study*—Summarise follow-up time (eg, average and total amount) n/a
Outcome data	15*	*Cohort study*—Report numbers of outcome events or summary measures over time
		Case-control study—Report numbers in each exposure category, or summary measures of exposure
		Cross-sectional study—Report numbers of outcome events or summary measures Pages 6 and 7 & Tables 1-4
Main results	16	(*a*) Pages 6 and 7 & Tables 1-4
		(*b*) Report category boundaries when continuous variables were categorized n/a
		(*c*) If relevant, consider translating estimates of relative risk into absolute risk for a meaningful time period – n/a
Other analyses	17	Report other analyses done—eg analyses of subgroups and interactions, and sensitivity analyses Pages 6 and 7 & Tables 1-4
**Discussion**		
Key results	18	Summarise key results with reference to study objectives Page 7
Limitations	19	Discuss limitations of the study, taking into account sources of potential bias or imprecision. Discuss both direction and magnitude of any potential bias Pages 3 and 9
Interpretation	20	Give a cautious overall interpretation of results considering objectives, limitations, multiplicity of analyses, results from similar studies, and other relevant evidence Pages 7-9
Generalisability	21	Discuss the generalisability (external validity) of the study results pages 7-9
**Other information**		
Funding	22	Give the source of funding and the role of the funders for the present study and, if applicable, for the original study on which the present article is based page 14

For peer review only

BMJ Open

Admission to Psychiatric Hospital for mental illnesses 2 years pre and post childbirth in Scotland: a health informatics approach to assessing mother and child outcomes.

Journal:	*BMJ Open*
Manuscript ID	bmjopen-2017-016908.R2
Article Type:	Research
Date Submitted by the Author:	07-Jul-2017
Complete List of Authors:	Langan, Julie; University of Glasgow, Institute of Health and Wellbeing<br>McLean, Gary; University of Glasgow, Institute of Health and Wellbeing<br>Martin, Daniel; Glasgow University, Institute of Health and Wellbeing<br>Cantwell, Roch; Leverndale Hospital, Perinatal Mental Health Services<br>Smith, Daniel; University of Glasgow, Institute of Health and Wellbeing
**Primary Subject Heading**:	Mental health
Secondary Subject Heading:	Health informatics, Health services research, Public health
Keywords:	mental illness, child outcomes, Mother and Baby Unit, psychiatric admissions

SCHOLARONE™
Manuscripts

For peer review only

Admission to Psychiatric Hospital for mental illnesses 2 years pre and post childbirth in Scotland: a health informatics approach to assessing mother and child outcomes.

Julie Langan Martin[1], Gary McLean[1], Daniel Martin[1], Roch Cantwell[2], Daniel J. Smith[1].

[1] Institute of Health and Wellbeing, College of Medical, Veterinary and Life Sciences, University of Glasgow, Mental Health and Wellbeing, Gartnavel Royal Hospital, 1055 Great Western Road, Glasgow G12 0XH

[2] Perinatal Mental Health Service & West of Scotland Mother & Baby Unit, Leverndale Hospital, 510 Crookston Road, Glasgow G53 7TU

Corresponding Author Julie Langan Martin Institute of Health and Wellbeing, College of Medical, Veterinary and Life Sciences, University of Glasgow, Mental Health and Wellbeing, Gartnavel Royal Hospital, 1055 Great Western Road, Glasgow G12 0XH Phone No: +44 (0)141 211 3927 Fax :+44 (0) 141 211 0356

Email: Julie.langan@glasgow.ac.uk

KEYWORDS: **mental illness, child outcomes, Mother and Baby Unit and hospital admission**

WORD COUNT: ABSTRACT: 300 MAIN TEXT: 2755

For peer review only

For peer review only - http://bmjopen.bmj.com/site/about/guidelines.xhtml

ABSTRACT

OBJECTIVE: To identify factors associated with: admission to a specialist Mother and Baby Unit (MBU), and the impact of perinatal mental illness on early childhood development using a data linkage approach in the 2 years pre and post childbirth.

METHODS: Scottish maternity records (SMR02) were linked to psychiatric hospital admissions (SMR04). 3,290 pregnancy-related psychiatric admissions for 1,730 women were assessed. To investigate factors associated with MBU admission, the group of mothers admitted to a MBU were compared to those admitted to general psychiatric wards. To assess the impact of perinatal mental illness on early child development, a pragmatic indicator for 'at potential risk of adversity', defined as a child who was recorded as requiring intensive treatment at any time under the health plan indicators (HPI) and/or who had no record of completing three doses of the 5 in 1 vaccine by 12 months was generated. Logistic regression models were used to describe the association between each variable and the risk of admission between those with a history of prior psychiatric admission and those without.

RESULTS: Women admitted to an MBU were significantly more likely to be admitted with non-affective psychosis (OR = 1.97, 95%CI 1.22-3.18), affective psychosis (OR = 2.44, 95%CI 1.37-4.33) and non-psychotic depressive episodes (OR = 1.93, 95%CI 1.42-2.63). They were less likely to come from deprived areas (OR: 0.68 95%CI 0.49-0.93). Women with a previous history of psychiatric admission were significantly more likely to be located in the two most deprived quintiles. Almost one third (29%) of children born to mothers with a pregnancy-related psychiatric admission were assessed as "at potential risk of adversity."

CONCLUSIONS: A health informatics approach has potential for improving understanding of social and clinical factors, which contribute to the outcomes of perinatal mental illness, as well as potential adverse developmental outcomes for offspring.

For peer review only - http://bmjopen.bmj.com/site/about/guidelines.xhtml

Strengths and limitations of this study

- Whole of Scotland childbirth and psychiatric admission data used for analyses, rather than local data only.
- Robust measure of socioeconomic status (Scottish Index of Multiple Deprivation) compared with other studies, which have used educational status.
- These analyses used only psychiatric admission data, rather than outpatient psychiatric attendances.
- Our definition of 'at potential risk of adversity' in offspring was a composite and pragmatic measure derived from the limited child health outcome data and there has been some inconsistency in the implementation of this across health boards.

For peer review only

For peer review only - http://bmjopen.bmj.com/site/about/guidelines.xhtml

BACKGROUND:

Good maternal mental health is important for normal childhood development[1]. Maternal mental illness can disrupt optimal parenting processes, and can adversely affect childhood development, especially emotional development[2,3,4]. In particular maternal mental illness may be associated with increased rates of childhood depression[5]and may also affect children's executive[6] and cognitive functioning[7]. Maternal depression both during pregnancy and in the post-natal period is common and is estimated to affect between 10 and 15% of women[8,9]. While the prevalence of postpartum psychosis is relatively low at 1/1000 births[10], it is known to be associated with severe adverse outcomes, including maternal suicide and infanticide[11].

The joint admission of mentally ill mothers and their infants was pioneered by Thomas Main in 1948[12]. Since then the UK, Australia and France have acted as leaders in this field[13,14]. Currently in the UK where possible, postpartum women (and those in later pregnancy) with severe mental illnesses such as psychosis or severe depressive disorder are admitted to a specialised Mother and Baby Unit (MBU). In the West of Scotland, women in the post-natal period who are the primary carer of their baby and thought to require psychiatric hospital admission are discussed with (and where possible assessed by) the local Consultant Perinatal Psychiatrist. Hospital admission is arranged if required. While most admissions are voluntary, MBUs can also accommodate women who require compulsory treatment under the Mental Health Act. The current Scottish Intercollegiate Guidelines Network (SIGN) clinical guidelines for the management of perinatal mood disorder reflect this[15]. It is recognised that women in the early postpartum period (0 to 6 weeks after childbirth), are at particularly elevated risk of requiring psychiatric admission[16,17]. In our previous study, we found that this risk remains elevated for up to 2 years post childbirth[16].

MBUs are highly specialised, expensive and limited resources, where expertise in both treatment of psychiatric disorders and childcare are required[18]. Although there are currently 15 in England and 2 in Scotland[19],access to this specialised service is poorer in many other High Income Countries (HICs; such as the US and Canada) and Low And Middle Income Countries (LAMICs) despite women appearing to be satisfied with this type of care[20]. Given the importance of adverse childhood experiences (ACEs) in future health[21,22], perinatal mental illness and the potential impact it has on offspring is a priority area for research and practice.

AIMS OF THE STUDY:

For peer review only - http://bmjopen.bmj.com/site/about/guidelines.xhtml

The aim of the study was to use a data linkage approach to investigate factors associated with admission to a specialist Mother and Baby Unit (MBU) and the risk to early childhood development in the context of a pregnancy related psychiatric admission.

MATERIALS AND METHODS:

We used a dataset from the Information Services Division (ISD) of NHS Scotland, which included all maternity records (SMR04) between 2005-2009, linked to all psychiatric hospital admission records (SMR02) between 2003-2011. This dataset has been described elsewhere[16]. However in brief, for each maternity record, any psychiatric admission was reported by week for the 104 weeks pre-childbirth and post-childbirth. Admission types were defined by ICD-10 codes: psychosis-only admissions included 'non-affective psychosis' (F20, F20.3, F20.5, F20.6, F20.8, F20.9, F21X, F22.0, F22.8, F22.9, F23.0, F23.1, F23.2, F23.3, F23.8, F23.9, F24X, F28X, F29X), 'affective psychosis' (F25.0, F25.1, F25.2, F25.9, F30.2, F31.2, F31.5, F32.3, F33.3) and 'postpartum psychosis' (F53.0, F53.1, F53.9); admissions due to a non-psychotic depressive episode included F32.0, F32.00, F32.01, F32.1, F32.10, F32.11, F32.2, F32.8, F32.9, F33.0, F33.00, F33.1, F33.10, F33.11, F33.2, F33.4, F33.8, F33.9. For the category of 'other admissions', we included all other ICD-10 codes recorded.

In total, 3,290 pregnancy-related psychiatric admissions for 1,730 women were assessed. For deliveries in 2005, psychiatric admissions between 2003 (two years before) and 2007 (two years after) were captured. Similarly, for deliveries in 2009, psychiatric admissions from 2007 (two years before) until 2011 (two years after) were captured. For each maternity record, any psychiatric admission was reported by week for the 2-year pre-and post-childbirth periods. Early childhood developmental outcomes (assessed by a Health Visitor at both 10-day and 6-8 week child-health checks) were also available. All statistical analyses were performed in STATA version 13.1[23].

To investigate factors associated with admission to the MBU, we compared the group of mothers admitted to one of two Scottish MBU units to those admitted to a general psychiatric ward up to 2 years post-childbirth on a range of sociodemographic characteristics (age, social deprivation and previous pregnancy). We included the geographical location (health board area) of the mother. We also compared differences in average length of stay. There are two MBUs in Scotland, the West of Scotland Mother and baby Unit (Leverndale Hospital, NHS Greater Glasgow and Clyde) and Mental Health Mother and Baby Unit, St John's Hospital, (West Lothian) where mothers are admitted with their baby in the post-partum period. For women admitted to a general psychiatric hospital this is generally without their baby.

For peer review only - http://bmjopen.bmj.com/site/about/guidelines.xhtml

To explore possible risk factors associated with previous psychiatric admission in the time period covered, we compared a group of mothers with a history of prior psychiatric admission to those without a history of admission two or more years prior to the index admission. We compared those with and without a previous admission by diagnosis, Scottish Index of Multiple Deprivation (SIMD) quintile, age group, length of stay, time of admission and whether the mother had a previous pregnancy. SIMD score was used as a measure of social deprivation. The SIMD identifies small areas of multiple deprivation (datazones) across Scotland by combining 38 indicators across 7 domains which are weighted. The domains include: current income (28%), employment (28%), health (14%), education (14%), geographic access to services (9%), crime (5%), and housing (2%) and are weighted based on evidence from Oxford University's Social Disadvantage Research Centre [24]. Individuals were divided into deprivation quintiles, depending on their deprivation score based on national averages.

We used logistic regression to describe the association between each variable and the risk of admission between those with a history of prior psychiatric admission and those without. Odds ratios were adjusted for age and deprivation quintile or for age only (deprivation quintile) and deprivation quintile (age only).

Finally, to assess the impact of perinatal mental illness on early child development after childbirth, we generated a pragmatic indicator for ''at potential risk of adversity'', defined as a child who was recorded as requiring intensive treatment at any time under the health plan indicators (HPI) and/or who had no record of completing three doses of the 5 in 1 vaccine by 12 months. The HPI is assessed at first visit, at six to eight weeks. For the HPI three possible outcomes can be recorded by the Health Visitor for the level of care required ('core', 'additional' and 'intensive'). It should be noted that results should be interpreted cautiously as there has been inconsistency in the implementation of the HPI across health boards and others have not validated this measure. Please also note that the time of assessment is not related to the time of first admission and is not an assessment of the admission care.

ETHICAL CONSIDERATIONS:

Ethical approval for this study was obtained by the NHS Privacy Advisory Committee (PAC) (XRB12089).

RESULTS:

For peer review only - http://bmjopen.bmj.com/site/about/guidelines.xhtml

In total 190 (11.0%) of this sample were admitted to a specialist MBU in Scotland. Table 1 highlights some important differences between MBU versus non-MBU admissions. Firstly, women admitted to an MBU were significantly more likely to be admitted with non-affective psychosis (OR = 1.97, 95%CI 1.22-3.18), affective psychosis (OR = 2.44, 95%CI 1.37-4.33) and non-psychotic depressive episodes (OR = 1.93, 95%CI 1.42-2.63). It is also notable that women admitted to an MBU were significantly more likely to live within affluent areas (and less likely to come from deprived areas) and in general more likely to be from an older age group (31-35, 36-40 and over 40) (Table 1). Women admitted to an MBU were largely from the NHS Greater Glasgow and Clyde Health Board area (54.2% overall).

Table 1 also shows that MBU admissions were significantly less likely to have had a previous pregnancy and less likely to have had a previous psychiatric admission. It also highlights important differences in length of stay for MBU versus non-MBU admissions. It shows that women admitted to an MBU were significantly less likely to have brief stays in hospital (of 10 days or less) but significantly more likely to have stays of more than 10 days, and more than twice as likely to have had a stay of 21-40 days or 40 days or more.

In total 562 (32.5%) of the 1,730 women had a history of prior psychiatric admission. Table 2 shows that this group were more likely to be admitted with a diagnosis of affective psychosis (7.3% vs. 2.6%, adjusted OR = 3.00, 95%CI 1.82-4.94) and non-affective psychosis (10.0% vs. 6.4%, adjusted OR = 1.55, 95%CI 1.07-2.25) but less likely to have an admission for a postpartum or non-psychotic depressive episode diagnosis. Those with a previous history of psychiatric admission were also significantly more likely to be located in the two most deprived quintiles and less likely to come from the two most affluent quintiles. Table 2 also shows they were less likely to be under 26 but significantly more likely to be from the 31-35,36-40 and over 40 age groups. No significant differences were identified for length of stay, previous pregnancy or time of admission.

Table 3 shows that 518 (29.9%) of offspring were defined as being “at potential risk of adversity” according to our criteria asassessed at the first Health Visitor visit after childbirth. Those at “potential risk” were more likely (although not statistically significantly) to have had a mother with non-affective psychosis (OR = 1.37, 95%CI 0.94-2.01) and significantly less likely to have a mother with a diagnosis of non-psychotic depression (OR = 0.65, 95%CI 0.51-0.83). It is notable that they were significantly more likely to come from the most deprived quintiles and less likely to be from more affluent quintiles. No differences were found by age of mother nor by place of first admission either

For peer review only - http://bmjopen.bmj.com/site/about/guidelines.xhtml

at a MBU or a non-MBU. Those children identified as "at potential risk of adversity" were more likely to have had a mother with a history of a previous psychiatric admission.

DISCUSSION:

The aim of this study was to assess the extent to which sociodemographic factors and perinatal mental illness affects treatment and outcomes for both mothers and children. We covered two main areas: admissions to an MBU and factors associated with children identified as at "potential risk of childhood adversity".

In this large Scottish sample, women admitted to a one of the two Scottish MBUs (compared to women admitted to general psychiatry wards) were significantly more likely be diagnosed with a psychotic illness (non-affective psychotic illness or affective psychotic illness) and less likely to be admitted with other illnesses. There was no difference for early post-partum psychosis admissions between MBU and non MBU. This is in keeping with the notion that MBU admission is reserved for women suffering from the most serious mental disorders such as postpartum psychosis, mania, major depressive episodes with psychosis or schizophrenia [18].

Women admitted to an MBU (compared to women admitted to general psychiatry wards) were more likely to live within affluent areas and in general more likely to be from an older age group (36-40 and over 40). It is possible that differences in socio-demographics of women accessing MBUs, might reflect a health inequality in terms of access to MBU admission. However this is a question, which requires further research.

To date literature exploring accessibility to MBUs is limited. There is one recent systematic review investigating outcomes for women admitted to a mother and baby unit[25]. However accessibility to an MBU was not reported. There are other studies on women admitted to an MBU, but they give only results on the socio-economic status of women and no objective marker of deprivation (such as SIMD) is usually included[25]. Studies investigating the link between deprivation and admission rate are limited. Some authors have described a link between psychiatric diagnoses, deprivation and admission rate[26] (in the general adult setting) while others have suggested a link between socio-economic status and admission to forensic psychiatry services[27].However further detailed investigation in this area is required.

For peer review only - http://bmjopen.bmj.com/site/about/guidelines.xhtml

In our study we found that women admitted to an MBU were more likely to have a longer average length of stay (40 plus days).This may be related to the need for a higher level of social functioning to ensure safe care for mothers and their babies on discharge from hospital. Further investigation comparing average length of stay in an MBU with particular focus on the cost effectiveness of MBUs may be warranted.

Several notable findings arose from our comparison of mothers of children identified as “at potential risk of adversity”. Firstly, almost one-third (29%) of children fell into this category. This rate is similar to that reported by others. For example, Whitmore and colleagues[28] reported that social services were involved in 31.6% of all admissions to their MBU in Birmingham and 10% of admissions resulted in separate discharges[28]. Howard and colleagues[29] reported that 23% of women were discharged with their babies under some form of social services supervision[29].

Secondly, our findings indicate that children in the “at potential risk of adversity” group were more likely to come from deprived locations, have mothers with a previous psychiatric admission and have had a mother admitted with a non-affective psychosis (schizophrenia). This finding is also similar to that by others [18,28-30]who reported that women with a diagnosis of schizophrenia were discharged separately more often than other groups. Potential risk factors associated with risk of separation are complex, but include: neonatal or infant medical problems or complications; maternal psychiatric disorder; paternal psychiatric disorder; maternal lack of good relationship with others; mother receipt of disability benefits; and low social class[30]. In particular schizophrenia, personality disorder, and poor social integration have all been related to poor clinical outcomes[31].

Successful parenting is complex, but may be partly associated with family stability and access to financial and social resources[32]. While it is recognised that mothers with severe mental illness, have specific needs relating to their children, their health and social care needs may not always be readily identified by healthcare professionals[33]. In cases of separate discharge, additional support should be made available to minimise distress. Therefore taken together, our findings suggest that there may be some benefit to identifying mothers at particularly high risk in terms of adverse developmental outcomes for their children, such as mothers living in more deprived communities who have a history of previous psychiatric admissions for schizophrenia.

For peer review only - http://bmjopen.bmj.com/site/about/guidelines.xhtml

STRENGTHS AND LIMITATIONS:

Strengths of this study include the completeness of the sample, which was obtained from record linkage for the whole of Scotland. However, some limitations in this work are acknowledged. Firstly, only psychiatric admission data were used, with no use of out-patient data. Our findings are therefore focused on the more severe end of the mental illness spectrum. Although we were able to determine if individuals had had a previous psychiatric admission, information about the number of previous admissions, family support, or access to Crisis teams were not available. The comparisons of women admitted to MBUs with women admitted to general psychiatric wards needs to be interpreted cautiously. This is particularly in relation to the timing of admissions in relation to childbirth, as women will only be admitted to MBUs in the very late stages of pregnancy and in the first year post-delivery. The general psychiatric admissions included admissions up to 2 years pre delivery and 2 years post-delivery and may therefore include women with long-term illnesses.

Furthermore, limited information about time and length stay at an MBU prevented any cost analysis being undertaken. Our definition of "at potential risk of adversity" in offspring was a composite and pragmatic measure derived from the limited child health outcome data which was available to us from record linkage. There has also been some inconsistency in the implementation of the HPI across health boards. A more detailed and comprehensive assessment of child development in this group of mothers is therefore warranted.

CONCLUSION:

In conclusion, this study found that a health informatics/data linkage approach has considerable potential for improving our understanding of the social and clinical factors, which contribute to perinatal mental illness in mothers in Scotland, as well as potential adverse developmental outcomes for their children. To date there has been no systematic assessment of the benefits (or adverse effects) of specialised MBUs in Scotland. Given the current political-economic climate and the importance of early intervention, further research in this area would be of benefit.

For peer review only - http://bmjopen.bmj.com/site/about/guidelines.xhtml

Table 1. Comparison of characteristics of women admitted either with their infant to a Mother-Baby Unit (MBU) or without their infant to a psychiatric ward (Non-MBU) (N=1729)

	MBU admission	Non-MBU admission	Odds ratio, adjusted by age and deprivation (95%CI)
	Number (%)	Number (%)	
Total	190 (11.0)	1,539 (89)	
**Diagnosis**			
Non affective psychosis	24 (12.6)	107 (7.0)	1.97 (1.22-3.18)
Affective psychosis	18 (9.5)	53 (3.4)	2.44 (1.37-4.33)
Post-partum (0-12 weeks) psychosis	18 (9.5)	87 (5.7)	1.56 (0.91-2.70)
Non psychotic depressive episodes	85 (44.7)	447 (29.0)	1.93 (1.42-2.63)
Any other diagnosis	130 (68.4)	1,292 (84.0)	0.45 (0.32-0.63)
**Deprivation Quintile**			
Most deprived (1)	64 (33.7)	656 (43.4)	0.68 (0.49-0.93)
2	34 (17.9)	336 (22.2)	0.76 (0.51-1.13)
3	28 (14.7)	246 (16.3)	0.88 (0.58-1.31)
4	37 (19.5)	169 (10.2)	2.00 (1.35-2.96)
Least deprived (5)	27 (14.2)	105 (6.9)	2.33 (1.49-3.65)
**Age group**			
Under 20	16 (8.4)	172 (11.2)	0.78 (0.45-1.34)
20-25	40 (21.1)	465 (30.2)	0.65 (0.45-0.95)
26-30	47 (24.7)	407 (26.4)	0.90 (0.63-1.29)
31-35	50 (26.3)	293 (19.0)	1.38 (0.97-1.96)
36-40	31 (16.3)	170 (11.0)	1.49 (1.03-1.58)
Over40	6 (3.2)	32 (2.1)	1.55 (0.63-3.80)
**Length of admission**			
Five days or less	35 (18.4)	640 (41.6)	0.34 (0.23-0.50)
6-10 days	25 (13.2)	320 (20.8)	0.59 (0.39-0.92)
11-20 days	42 (22.1)	274 (17.8)	1.27 (0.86-1.85)
21-40 days	49 (25.8)	172 (11.2)	2.51 (1.73-3.63)
40 plus days	39 (20.4)	133 (8.6)	2.48 (1.65-3.71)
**Geographical Location**			
Greater Glasgow and Clyde	103 (54.2)	389 (25.3)	3.50 (2.57-4.76)
Lothian	55 (29.0)	169 (11.0)	3.30 (2.32-4.96)
Lanarkshire	12 (6.3)	169 (11.0)	0.54 (0.29-1.00)
Any other Health Board	20 (10.5)	812 (52.7)	0.02 (0.01 to 0.03)
Had previous pregnancy	95 (50.0)	935 (60.8)	0.57 (0.41-0.78)
History of prior psychiatric admission	43 (22.6)	519 (33.7)	0.57 (0.40-0.83)
Time of first admission			
Post-partum (0-12-weeks)	102 (53.7)	125 (8.1)	11.96 (8.4-16.8)

Note: women could have more than one diagnosis so numbers are greater than the number of women. In total 27 women had no Scottish Index of Multiple Deprivation (SIMD) quintile assigned due to missing or incorrect postcode and were excluded from the regression analysis for all variables

For peer review only - http://bmjopen.bmj.com/site/about/guidelines.xhtml

Table 2. Comparison of characteristics of women with and without a previous psychiatric admission prior to the index admission (N=1720).

	**Previous admission** Number (% of total)	**No previous admission** Number (% of total)	**Odds ratio adjusted by age and deprivation (95%CI)**
Total	562 (32.5)	1,168 (67.5)	
**Diagnosis**			
Non affective psychosis	56 (10.0)	75 (6.4)	1.55 (1.07-2.25)
Affective psychosis	41 (7.3)	30 (2.6)	3.00 (1.82-4.94)
Post-partum psychosis	57 (10.1)	170 (14.6)	0.36 (0.20-0.64)
Non psychotic depressive episodes	137 (24.4)	395 (33.8)	0.63 (0.50-0.79)
Any other diagnosis	450 (80.1)	973 (83.3)	0.78 (0.60-1.02)
**Deprivation Quintile**			
Most deprived	251 (45.7)	470 (40.7)	1.31 (1.07-1.61)
2	131 (23.9)	239 (20.7)	1.11 (0.87-1.42)
3	85 (15.5)	189 (16.4)	0.90 (0.68-1.20)
4	54 (9.8)	152 (13.2)	0.68 (0.49-0.95)
Least deprived	28 (5.1)	104 (9.0)	0.48 (0.31-0.75)
**Age group**			
Under 20	37 (6.6)	152 (13.0)	0.44 (0.30-0.64)
20-25	146 (26.0)	359 (30.7)	0.78 (0.62-0.98)
26-30	160 (28.5)	294 (25.2)	1.19 (0.95-1.50)
31-35	126 (22.4)	217 (18.6)	1.31 (1.01-1.68)
36-40	80 (14.2)	121 (10.4)	1.45 (1.07-1.97)
Over40	13 (2.3)	25 (2.1)	1.01 (0.50-2.03)
**Length of stay**			
Five days or less	203 (36.1)	472 (40.4)	0.87 (0.70-1.09)
6-10 days	112 (19.9)	234 (20.0)	0.99 (0.76-1.28)
11-20 days	104 (18.5)	212 (18.2)	0.95 (0.73-1.24)
21-40 days	71 (12.6)	150 (12.8)	0.99 (0.73-1.26)
40 plus days	72 (12.8)	100 (8.6)	1.50 (1.07-2.09)
Had previous pregnancy	359 (63.9)	671 (57.5)	1.03 (0.82-1.29)
Time of first admission			
Post-partum (0-12-weeks	57 (10.1)	170 (14.6)	0.71 (0.48-1.02)

Note: women could have more than one diagnosis so numbers are greater than the number of women. In total 27 women had no Scottish Index of Multiple Deprivation (SIMD) quintile assigned due to missing or incorrect postcode and were excluded from the regression analysis for all variables

For peer review only - http://bmjopen.bmj.com/site/about/guidelines.xhtml

Table 3. Characteristics of mothers with children defined as being at 'potential risk of adversity' (N=1720)

	At potential risk of adversity	Not at potential risk of adversity	Odds ratio adjusted by age and deprivation (95%CI)
	Number (% of total)	Number (% of total)	
**Total**	518 (29.9)	1,212 (70.1)	
MBU Stay	58 (30.4)	133 (69.6)	1.18 (0.84-1.65)
General Ward	459 (29.8)	1,079 (70.2)	1
**Diagnosis**			
Non affective psychosis	49 (9.5)	82 (6.7)	1.37 (0.94-2.01)
Affective psychosis	22 (4.3)	49 (4.0)	1.24 (0.73-2.12)
Post-partum psychosis	21 (4.1)	84 (6.9)	0.62 (0.37-1.03)
Non psychotic depressive episodes	127 (24.5)	405 (33.4)	0.65 (0.51-0.83)
Any other diagnosis	426 (82.2)	997 (82.3)	0.93 (0.70-1.22)
**Deprivation Quintile**			
Most deprived	274 (54.0)	448 (37.5)	1.88 (1.52-2.32)
2	112 (22.1)	227 (21.5)	1.03 (0.80-1.33)
3	61 (12.0)	214 (17.9)	0.61 (0.45-0.84)
4	36 (7.1)	169 (14.1)	0.46 (0.32-0.68)
Least deprived	24 (7.3)	108 (9.3)	0.51 (0.32-0.81)
**Age group**			
Under 20	61 (11.8)	128 (10.6)	1.02 (0.73-1.45)
20-25	154 (29.7)	351 (29.0)	0.91 (0.72-1.15)
26-30	154 (29.7)	300 (24.8)	1.32 (0.98-1.67)
31-35	90 (17.4)	253 (20.9)	0.90 (0.69-1.17)
36-40	47 (9.1)	154 (12.7)	0.73 (0.51-1.03)
Over40	12 (2.3)	26 (2.2)	1.15 (0.56-2.33)
**Length of stay**			
Five days or less	213 (41.1)	456 (37.6)	1.16 (0.93-1.44)
6-10 days	103 (19.9)	244 (20.1)	0.91 (0.70-1.19)
11-20 days	93 (18.0)	223 (18.4)	0.98 (0.73-1.29)
21-40 days	48 (11.2)	166 (13.7)	0.84 (0.60-1.17)
40 plus days	51 (9.9)	123 (10.2)	0.98 (0.68-1.40)
Had previous pregnancy	319 (61.6)	711 (58.9)	1.11 (0.89-1.40)
History of prior psychiatric admission	215 (41.5)	347 (28.6)	1.76 (1.41-2.21)
Time of first admission			
Post-partum (0-12-weeks	73 (14.1)	154 (12.7)	1.31 (0.96-1.79)

Note: women could have more than one diagnosis so numbers are greater than the number of women. In total 27 women had no Scottish Index of Multiple Deprivation (SIMD) quintile assigned due to missing or incorrect postcode and were excluded from the regression analysis for all variables
Note: ''at potential risk of adversity'', is defined as a child who was recorded as requiring intensive treatment at any time under the health plan indicators (HPI) and/or who had no record of completing three doses of the 5 in 1 vaccine by 12 months. HPI is assessed at first visit and at six to eight weeks.

For peer review only - http://bmjopen.bmj.com/site/about/guidelines.xhtml

Contributor ship statement:
GM undertook the data analyses. JLM and DJM wrote the initial draft of the paper. GM, RC and DJM were involved in the drafting of the paper

Competing interests:
All authors report no competing interests.

Funding:
This project was funded by a CSO Health Informatics Call Grant

Data sharing statement:
Data included in the study are available to the authors only

For peer review only

For peer review only - http://bmjopen.bmj.com/site/about/guidelines.xhtml

REFERENCES:

1 Stein A, Pearson RM, Goodman SH, Rapa E, Rahman A, McCallum M, Howard LM, ParianteCMEffects of perinatal mental disorders on the fetus and child.Lancet. 2014 Nov 15;384(9956):1800-19. doi: 10.1016/S0140-6736(14)61277-0. Epub 2014 Nov 14.

2 Milgrom J, Ericksen J, Sved-Williams A. Chapter Impact of Parental Psychiatric Illness on Infant Development in Sutter-Dallay A-L, Glangeaud-Freudenthal NM-C, Guedeney A, Riecher-Rössler A (eds) Joint care of parents and infants in perinatal psychiatry. 2016, Springer,47-78.

3 Leis JA, Heron J, Stuart EA, Mendelson T. Associations between maternal mental health and child emotional and behavioral problems: does prenatal mental health matter? J Abnorm Child Psychol 2013; 42: 161–71

4 Yarrow L.J., MacTurk R.H., Vietze P.M., McCarthy M.E., Klein R.P., McQuinston S. Development course of parental stimulation and its relationship to mastery motivation during infancy. Developmental Psychology. 1984;20:492–503.

5 Murray L., Halligan S.L., & Cooper P.J. Effects of postnatal depression on mother-infant interactions, and child development In Wachs B.G., editor. (Ed.), 2010 Handbook of infant development (pp. 192–220). Malden: Wiley-Blackwell.

6 Hughes C, Roman G, Hart MJ, Ensor R. Does maternal depression predict young children's executive function? - a 4-year longitudinal study. J Child Psychol Psychiatry. 2013 Feb;54(2):169-77. doi: 10.1111/jcpp.12014. Epub 2012 Nov

7 Sutter-Dallay AL, Murray L, Dequae-Merchadou L, Glatigny-Dallay E, Bourgeois ML, Verdoux H.A prospective longitudinal study of the impact of earlypostnatalvs. chronicmaternaldepressive symptoms on child development. Eur Psychiatry.2011 Nov;26(8):484-9. doi: 10.1016/j.eurpsy.2010.05.004. Epub 2010 Jul 10

8 O'Hara MW, Swain AM Rates and risk of postpartum depression: a meta-analysis Int Rev Psychiatry, 1996, 8 :37–54

9 Pearlstein T, Howard M, Salisbury A, Zlotnick C. Postpartum depression Am J ObstetGynecol, 2009. 200: 357–364

10 Terp IM, Mortensen PB. Post-partum psychoses. Clinical diagnoses and relative risk of admission after parturition. Br J Psychiatry 1998;172:521–6.

11 Wisner KL, Peindl K, Hanusa BH. Symptomatology of affective and psychotic illnesses related to childbearing. J Affect Disord 1994;30:77–87. doi:10.1016/0165-0327(94)90034-5

12 Brockington IF. Oxford: Oxford University Press; 1996. Motherhood and Mental Health

13 Brockington I, Butterworth R, Glangeaud-Freudenthal N.An international position paper on mother-infant (perinatal) mental health, with guidelines for clinical practice.Archives of Women's Mental Health. 2017;20(1):113-120. doi:10.1007/s00737-016-0684-7.

For peer review only - http://bmjopen.bmj.com/site/about/guidelines.xhtml

14 Cazas O, Glangeaud-Freudenthal NM-C. The history of Mother-Baby Units (MBUs) in France and Belgium and of the French version of the Marcé checklist. *Archives of Women's Mental Health*. 2004;7(1):53-58. doi:10.1007/s00737-003-0046-0.

15 SIGN 127 Management of perinatal mood disorders Available at: **http://www.sign.ac.uk/pdf/sign127.pdf**Accessed 01/03/2017

16 Langan-Martin, J., McLean, G., Cantwell, R., and Smith, D. J.Admission to psychiatric hospital in the early and late post-partum periods: Scottish national linkage study.*BMJ Open*, 2016 6(1), e008758. (doi:10.1136/bmjopen-2015-008758)(MID:26733566)

17 Munk-Olsen T, Laursen TM, Pedersen CB et al New parents and mental disorders: a population-based registered study. JAMA 2006 296:2582–2589

18 Glangeaud-Freudenthal NM, Howard LM, Sutter-Dallay AL.Treatment - mother-infant inpatient units.Best Pract Res ClinObstetGynaecol. 2014 Jan;28(1):147-57. doi: 10.1016/j.bpobgyn.2013.08.015. Epub 2013 Sep 3.

19 Royal College of Psychiatrists Quality Network for Perinatal Mental Health Services (2016).http://www.rcpsych.ac.uk/pdf/Annual%20Inpatient%20Report%20Final%20Draft.pdf Accessed 16/03/2017

20 Neil S, Sanderson H, Wieck A (2006) A satisfaction survey of women admitted to a psychiatric mother and baby unit in the northwest of England. Arch WomensMent Health 2006: 9:109–112.

21 Bellis MA, Hughes K, Leckenby N Hardcastle KA, Perkins C, Lowey H. Measuring mortality and the burden of adult disease associated with adverse childhood experiences in England: a national survey. J Public Health (Oxf). 2015; 37(3):445-54. doi: 10.1093/pubmed/fdu065. Epub 2014 Aug 30.

22 Felitti VJ, Anda RF, Nordenberg D, Williamson DF, Spitz AM, Edwards V, Koss MP, Marks JS. Relationship of childhood abuse and household dysfunction to many of the leading causes of death in adults. The Adverse Childhood Experiences Study. Am J Prev Med. 1998;14:245–258

23 STATA: https://www.stata.com/stata-news/news28-4/stata13.1/

24 SIMD Scotland http://simd.scotland.gov.uk/publication-2012/introduction-to-simd-2012/overview-of-the-simd/what-is-the-simd/ Accessed 09/05/2017

25 Gillham R, Wittkowski A. Outcomes for women admitted to a mother and baby unit: a systematic review. *International Journal of Women's Health*. 2015;7:459-476. doi:10.2147/IJWH.S69472.

26 Harrison J, Barrow S, Creed F.Social deprivation and psychiatric admission rates among different diagnostic groups. Br J Psychiatry. 1995 Oct;167(4):456-62.

27 Coid J, Kahtan N, Cook A, Gault S, JarmanB.Predicting admission rates to secure forensic psychiatry services. Psychol Med. 2001 Apr;31(3):531-9.

28 Whitmore J, Heron J, WainscottG.Predictors of parenting concern in a Mother and Baby Unit over a 10-year period. Int J Soc Psychiatry. 2011 Sep;57(5):455-61. doi: 10.1177/0020764010365412. Epub 2010 Apr 13.

For peer review only - http://bmjopen.bmj.com/site/about/guidelines.xhtml

29 Howard LM, Thornicroft G, Salmon M, Appleby LPredictors of parenting outcome in women with psychotic disorders discharged from mother and baby units. Acta Psychiatr Scand. 2004 Nov;110(5):347-55.

30 Glangeaud NM Sutter-Dallay AL, Thieulin AC, Dagens V, Zimmermann MA, Debourg A, Amzallag C, Cazas O, Cammas R, Klopfert ME, Rainelli C, Tielemans P, Mertens C, Maron M, Nezelof S, Poinso F. Predictors of infant foster care in cases of maternal psychiatric disorders
Soc Psychiatry Psychiatr Epidemiol 2013: 48:553–561

31 Glangeaud NM, Sutter AL, Thieulin AC, Dagens-Lafont V, Zimmermann MA, Debourg A, Massari B, Cazas O, Cammas R, Rainelli C, Poinso F, Maron M, Nezelof S, Ancel PY, Khoshnood B.European Psychiatry 2011: 26: 215–223 doi:10.1016/j.eurpsy.2010.03.006

32Abel KM, Webb RT, Salmon MP, Wan MW, Appleby L. Prevalence and predictors of parenting outcomes in a cohort of mothers with schizophrenia admitted for joint mother and baby psychiatric care in England. J Clin Psychiatry 2005: 66:781–789.

33 Howard LM, Hunt K The needs of mothers with severe mental illness: a comparison of assessments of needs by staff and patients. Arch Womens Ment Health 2008: 11:131–136.

For peer review only

STROBE Statement—checklist of items that should be included in reports of observational studies

	Item No	Page Number discussed
**Title and abstract**	1	(*a*) Page 1
		(*b*) Page 2
**Introduction**		
Background/rationale	2	Page 4
Objectives	3	Page 4
**Methods**		
Study design	4	Page 5
Setting	5	Page 5
Participants	6	(*a*) *Cross-sectional study*—Page 5
		(*b*) *No matching*
Variables	7	Page 5
Data sources/ measurement	8*	Page 5
Bias	9	Page 9
Study size	10	Page 5
Quantitative variables	11	Page 5
Statistical methods	12	(*a*) Page 5
		(*b*) Describe any methods used to examine subgroups and interactions Page 5
		(*c*) Explain how missing data were addressed Page 5
		(*d*) *Cross-sectional study*—If applicable, describe analytical methods taking account of sampling strategy Page 5
		(*e*) Describe any sensitivity analyses: n/a

Continued on next page

For peer review only

**Results**		
Participants	13*	(a) Page 5
		(b) n/a
		(c) N/a
Descriptive data	14*	(a) Page 6
		(b) n/a
		(c) *Cohort study*—Summarise follow-up time (eg, average and total amount) n/a
Outcome data	15*	*Cohort study*—Report numbers of outcome events or summary measures over time
		Case-control study—Report numbers in each exposure category, or summary measures of exposure
		Cross-sectional study—Report numbers of outcome events or summary measures Pages 6 and 7 & Tables 1-4
Main results	16	(*a*) Pages 6 and 7 & Tables 1-4
		(*b*) Report category boundaries when continuous variables were categorized n/a
		(*c*) If relevant, consider translating estimates of relative risk into absolute risk for a meaningful time period – n/a
Other analyses	17	Report other analyses done—eg analyses of subgroups and interactions, and sensitivity analyses Pages 6 and 7 & Tables 1-4
**Discussion**		
Key results	18	Summarise key results with reference to study objectives Page 7
Limitations	19	Discuss limitations of the study, taking into account sources of potential bias or imprecision. Discuss both direction and magnitude of any potential bias Pages 3 and 9
Interpretation	20	Give a cautious overall interpretation of results considering objectives, limitations, multiplicity of analyses, results from similar studies, and other relevant evidence Pages 7-9
Generalisability	21	Discuss the generalisability (external validity) of the study results pages 7-9
**Other information**		
Funding	22	Give the source of funding and the role of the funders for the present study and, if applicable, for the original study on which the present article is based page 14

For peer review only

For peer review only - http://bmjopen.bmj.com/site/about/guidelines.xhtml
